# The scaffold-dependent function of RIPK1 in dendritic cells promotes injury-induced colitis

**DOI:** 10.1038/s41385-021-00446-y

**Published:** 2021-08-30

**Authors:** Kenta Moriwaki, Christa Park, Kazuha Koyama, Sakthi Balaji, Kohei Kita, Ryoko Yagi, Sachiko Komazawa-Sakon, Manami Semba, Tatsuya Asuka, Hiroyasu Nakano, Yoshihiro Kamada, Eiji Miyoshi, Francis K. M. Chan

**Affiliations:** 1 Department of Biochemistry, Toho University School of Medicine, Ota-ku, Tokyo Japan; 2 Department of Immunology, Duke University School of Medicine, Durham, NC USA; 3 Department of Pathology, Immunology and Microbiology Program, University of Massachusetts Medical School, Worcester, MA USA; 4 Department of Molecular Biochemistry and Clinical Investigation, Osaka University Graduate School of Medicine, Suita, Osaka Japan; 5 Department of Biological Science and Technology, Faculty of Advanced Engineering, Tokyo University of Science, Katsushika-Ku, Tokyo Japan; 6 Present Address: AbbVie Bioresearch Center, Worcester, MA USA; 7 Present Address: Department of Advanced Metabolic Hepatology, Osaka University Graduate School of Medicine, Suita, Osaka Japan

## Abstract

Receptor interacting protein kinase 1 (RIPK1) is a cytosolic multidomain protein that controls cell life and death. While RIPK1 promotes cell death through its kinase activity, it also functions as a scaffold protein to promote cell survival by inhibiting FADD-caspase 8-dependent apoptosis and RIPK3-MLKL-dependent necroptosis. This pro-survival function is highlighted by excess cell death and perinatal lethality in *Ripk1*^−/−^ mice. Recently, loss of function mutation of *RIPK1* was found in patients with immunodeficiency and inflammatory bowel diseases. Hematopoietic stem cell transplantation restored not only immunodeficiency but also intestinal inflammatory pathology, indicating that RIPK1 in hematopoietic cells is critical to maintain intestinal immune homeostasis. Here, we generated dendritic cell (DC)-specific *Ripk1*^−/−^ mice in a genetic background with loss of RIPK1 kinase activity and found that the mice developed spontaneous colonic inflammation characterized by increased neutrophil and Ly6C^+^ monocytes. In addition, these mice were highly resistant to injury-induced colitis. The increased colonic inflammation and the resistance to colitis were restored by dual inactivation of RIPK3 and FADD, but not by inhibition of RIPK3, MLKL, or ZBP1 alone. Altogether, these results reveal a scaffold activity-dependent role of RIPK1 in DC-mediated maintenance of colonic immune homeostasis.

## Introduction

Receptor interacting protein kinase 1 (RIPK1) is a cytosolic serine/threonine kinase that functions downstream of various cell surface immune-related receptors such as tumor necrosis factor receptor 1 (TNFR1)^1^. RIPK1 consists of a kinase domain (KD) and a death domain (DD) at the N- and C-termini respectively. In addition, RIPK1 harbors the RIP homotypic interaction motif (RHIM) in the intermediate region. Upon tumor necrosis factor (TNF) stimulation, RIPK1 is recruited to TNFR1 through homotypic DD interaction. In the membrane-bound TNFR1 complex, called complex I, RIPK1 is modified by K63- and M1-ubiquitination by the action of cellular inhibitor of apoptosis 1 (cIAP1) and linear ubiquitin chain assembly complex (LUBAC)^2^. These ubiquitin chains on RIPK1 act as a scaffold to recruit various signaling kinases such as an inhibitor of κB kinase (IKK) α/β, IKKε, transforming growth factor β activated kinase 1 (TAK1), and TANK binding kinase 1 (TBK1)^3^. These kinases directly or indirectly phosphorylate RIPK1 to inhibit its activation. In addition, IKKs phosphorylate inhibitor of κB (IκB), leading to NF-κB-dependent expression of pro-survival molecules such as cellular FLICE inhibitory protein (cFLIP)^4^. When these kinases are inhibited, RIPK1 interacts with FADD and caspase 8 to induce RIPK1-dependent apoptosis. In addition, when the expression of pro-survival proteins is inhibited by, for example, cycloheximide, cells can undergo RIPK1-independent apoptosis. This RIPK1-indepenent apoptosis is potentiated by loss of RIPK1 through diminished induction of pro-survival gene expression.

Besides apoptosis, RIPK1 also plays a role in an alternative cell death modality termed necroptosis, a regulated form of cell necrosis mediated by RIPK3 and the downstream effector mixed lineage kinase domain-like pseudokinase (MLKL)^5^. During TNFR1-induced necroptosis, RIPK1 interacts with RIPK3 via homotypic RHIM interaction in a RIPK1 kinase activity-dependent manner. The interaction promotes activation of RIPK3 and the conversion of the RIPK1-RIPK3 complex into an amyloid signaling complex called the necrosome^6,7^. In addition to RIPK1, RIPK3 can also be activated by interaction with other RHIM-containing proteins such as the toll-like receptor adaptor protein TRIF and the cytosolic Z-RNA receptor ZBP1^8,9^. Paradoxically, RIPK1 has been discovered to inhibit TRIF-RIPK3 and ZBP1-RIPK3 necrosomes in a RHIM-dependent and kinase activity-independent manner under certain conditions^10–13^. Therefore, the scaffold and the kinase functions of RIPK1 promote cell survival and cell death, respectively.

It has been demonstrated by various genetic and chemical approaches that RIPK1 kinase activity is involved in the development of infectious and non-infectious diseases^14^. Since *Ripk1* kinase-dead mutant knock-in mice develop and grow normally without any obvious abnormality, RIPK1 has garnered attention as a therapeutic target for various inflammatory diseases^15^. In contrast, *Ripk1*^−/−^ mice develop systemic inflammation and an early postnatal lethality due to aberrant apoptosis and necroptosis in multiple tissues^10,11,16^. These phenotypes are rescued by concomitant blockade of apoptosis through deletion of either FADD or caspase 8 and necroptosis through deletion of either RIPK3 or MLKL. Recently, biallelic loss of function mutations of *RIPK1* was found in patients who suffered from primary immunodeficiency and peripheral inflammation^17–19^. One of the most common pathologies observed in these patients was early-onset inflammatory bowel disease. Interestingly, hematopoietic stem cell transplantation resolved clinical symptoms of inflammatory bowel disease as well as reduced the frequency of recurrent infection, highlighting that RIPK1 in hematopoietic cells is critical to maintain immune homeostasis and protect autoinflammation in peripheral tissues^17^. Although cell type-specific functions of RIPK1 have been investigated using conditional *Ripk1* knockout mice^20–24^, the immune cell type in which RIPK1 functions to protect against inflammatory bowel disease is unknown at present.

Dendritic cells (DCs) are central immune effectors that recognize foreign antigens and control acquired and innate immune responses^25^. We previously reported that RIPK3 promotes cytokine production and tissue repair in a kinase activity-independent manner in DCs during injury-induced colitis^26–28^. By contrast, it remains unknown whether RIPK1 in DCs plays a similar role in intestinal inflammation. Here, we utilized *Ripk1* kinase-dead floxed mice and investigated the differential roles of RIPK1 kinase and scaffold functions in DCs in intestinal immune homeostasis. We found that DC-specific deletion of RIPK1 caused FADD-dependent spontaneous colonic inflammation characterized by increased neutrophil and Ly6C^+^ monocytes. Despite the elevated basal colonic inflammation, loss of RIPK1 in DCs surprisingly rendered mice resistant to injury-induced colitis. These results demonstrate that RIPK1 in DCs has a critical impact on intestinal immune homeostasis.

## Results

### DC-specific deletion of RIPK1 causes splenic inflammation independent of RIPK1 kinase activity

**Fig. 1:**
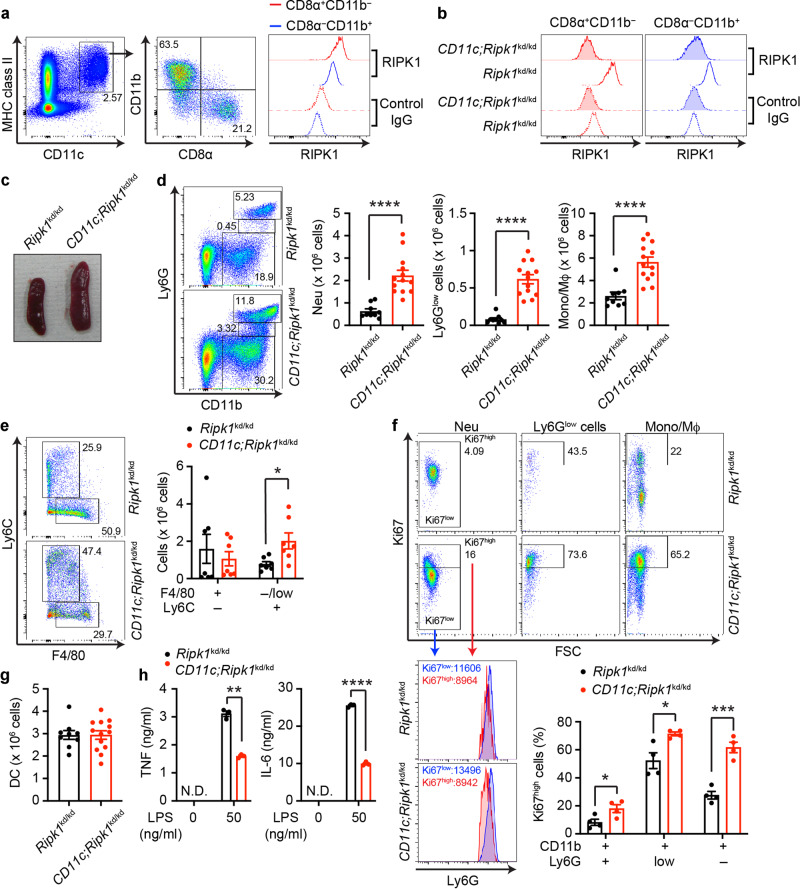
DC-specific RIPK1 deletion causes inflammation in the absence of RIPK1 kinase activity. **a**, **b** Splenocytes of *Ripk1*^kd/kd^ (**a**, **b**) and CD11c;*Ripk1*^kd/kd^ mice (**b**) were subjected to RIPK1 intracellular staining. Representative results of flow cytometry are shown (*n* = 4 mice). CD45^+^ cells are presented (**a**, the most left panel). **c** A representative picture of the spleen from mice with the indicated genotypes is shown. **d** The number of Ly6G^+^ neutrophils (Neu), Ly6G^−^ monocytes/macrophages (Mono/Mφ), and Ly6G^low^ cells among CD45^+^ CD3^-^B220^−^CD11c^−^CD11b^+^ cells in the spleen is shown (*n* = 9 for *Ripk1*^kd/kd^ mice and *n* = 13 for CD11c;*Ripk1*^kd/kd^ mice). **e** The number of F4/80^+^Ly6C^−^ macrophages and F4/80^-/low^Ly6C^+^ monocytes among CD45^+^ CD3^−^CD19^−^CD11c^−^CD11b^+^ Ly6G^−^ Mono/Mφ in the spleen is shown (*n* = 7 per each genotype). **f** Splenocytes were subjected to Ki67 staining. Percentages of Ki67^high^ cells among Ly6G^+^ neutrophils, Ly6G^−^ Mono/ Mφ, and Ly6G^low^ cells are shown (*n* = 4 mice per each genotype). Mean fluorescent intensity of Ly6G is shown in the histogram. **g** The number of CD11c^+^ DCs among CD45^+^ CD3^-^B220^−^ cells in the spleen is shown (*n* = 9 for *Ripk1*^kd/kd^ mice and *n* = 13 for CD11c;*Ripk1*^kd/kd^ mice). **h** Concentrations of TNF and IL-6 produced by BMDCs treated with LPS for 6 h are shown (*n* = 3 per each genotype. A representative result from two independent experiments is shown.). N.D.: not detected. Mice from the intercross between *Ripk1*^kd/kd^ and CD11c;*Ripk1*^kd/kd^ mice were used. Results are mean ± SEM. *p < 0.05, **p < 0.01, ***p < 0.001, ****p < 0.0001 (unpaired *t* test with Welch’s correction).

RIPK1 controls multiple signaling pathways through kinase activity-dependent and -independent functions. To delineate the kinase activity-dependent and -independent roles in DCs, we crossed *Ripk1* kinase-dead mutant knock-in mice (*Ripk1*^kd/kd^)^29^, in which exon 4 was flanked by *loxP* sequences, with CD11c-Cre transgenic mice (CD11c;*Ripk1*^kd/kd^ mice). In the spleen, DCs are mainly divided into two subsets: CD8α^+^ CD11b^-^ conventional DC1 (cDC1) and CD8α^-^CD11b^+^ cDC2^25^. RIPK1 expression was slightly higher in cDC1 than in cDC2 (Fig. 1a). In both subsets, RIPK1 was deleted in CD11c;*Ripk1*^kd/kd^ mice (Fig. 1b). Previous reports showed that *Ripk1*^kd/kd^ mice grow normally and did not show any overt abnormalities at steady state^29,30^. We confirmed normal spleen size and splenic immune cell populations in *Ripk1*^kd/kd^ mice (Fig. S1a and S1b). In contrast, we observed splenomegaly and a significant increase of the number of CD11b^+^ Ly6G^high^ neutrophils and CD11b^+^ Ly6G^-^ monocytes/macrophages (Mono/Mφ) in the spleen of CD11c;*Ripk1*^kd/kd^ mice (Fig. 1c, d). CD11b^+^ Ly6G^-^ Mono/Mφ are divided into mainly two populations: F4/80^+^ macrophages and Ly6C^+^ monocytes in the spleen (Fig. 1e). We found that Ly6C^+^ monocytes were significantly increased in CD11c;*Ripk1*^kd/kd^ mice (Fig. 1e). In addition, we noticed that CD11b^+^ Ly6G^low^ cells were significantly increased in CD11c;*Ripk1*^kd/kd^ mice (Fig. 1d). Staining of the proliferation marker Ki67 revealed that many of CD11b^+^ Ly6G^low^ cells highly expressed Ki67, especially in CD11c;*Ripk1*^kd/kd^ mice, which indicates that these cells were highly proliferative (Fig. 1f and S1c). Among CD11b^+^ Ly6G^high^ neutrophils and CD11b^+^ Ly6G^-^ Mono/Mφ, Ki67^high^ population was also significantly increased in CD11c;*Ripk1*^kd/kd^ mice (Fig. 1f and S1c). Consistent with the high expression of Ki67 in CD11b^+^ Ly6G^low^ cells, Ki67^high^ neutrophils showed lower expression of Ly6G than Ki67^low^ neutrophils (Fig. 1f, histogram). These results suggest that the enhanced cell proliferation contributed to the increase of neutrophils and Mono/Mφ.

Although deletion of RIPK1 sensitizes cells to apoptosis and necroptosis^10,11,16^, the number of splenic DCs did not alter in CD11c;*Ripk1*^kd/kd^ mice (Fig. 1g and S1d). Cell proliferation of splenic DCs was not changed in CD11c;*Ripk1*^kd/kd^ mice (Fig. S1e). Similar to the previous observation in mouse embryonic fibroblasts^31^, LPS-induced cytokine expression in BMDCs was reduced by RIPK1 deletion (Fig. 1h). These results suggest that RIPK1-deficient DCs are functionally impaired.

Similar increase of neutrophils, Mono/Mφ, and CD11b^+^ Ly6G^low^ cells were observed in the BM of CD11c;*Ripk1*^kd/kd^ mice, although the difference was smaller than that in the spleen (Fig. S2a). In addition, DC number was not changed in the BM of CD11c;*Ripk1*^kd/kd^ mice (Fig. S2a). A clear difference from the spleen was that Ki67^high^ population among any of these cell types was not increased in the BM of CD11c;*Ripk1*^kd/kd^ mice (Fig. S2b), suggesting a tissue-specific effect on the increase of myeloid cells upon DC-specific RIPK1 deletion.

### DC-specific deletion of RIPK1 reduces the number of DCs in the colon

**Fig. 2:**
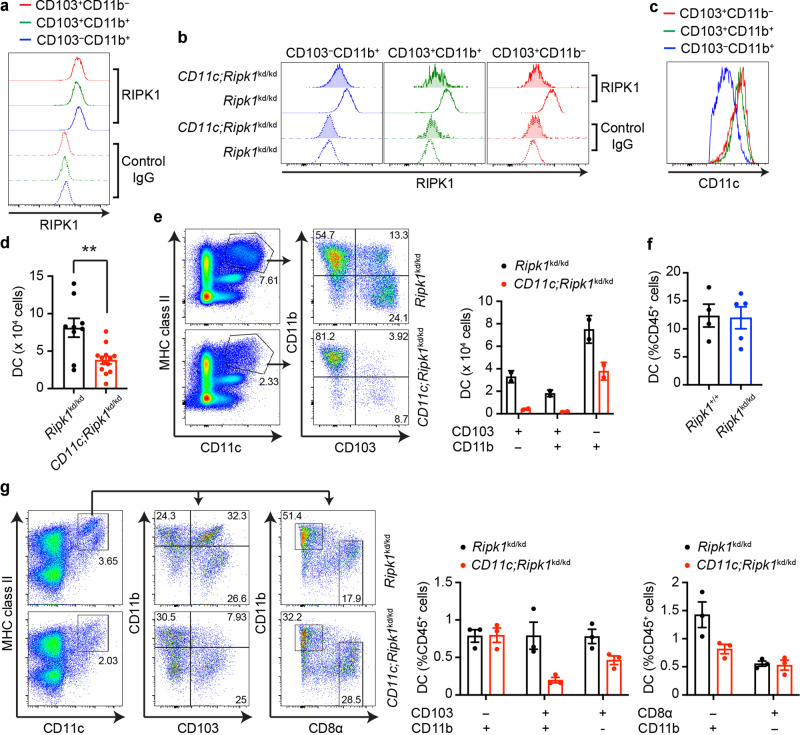
DC-specific RIPK1 deletion causes spontaneous decrease of DCs in the colon. **a**, **b** Colonic lamina propria cells isolated from of *Ripk1*^kd/kd^ (**a**, **b**) and CD11c;*Ripk1*^kd/kd^ mice (**b**) were subjected to RIPK1 intracellular staining. Representative results of flow cytometry are shown (*n* = 4 per each genotype). CD45^+^ CD11c^+^ MHC class II^+^ cells are presented. **c** A representative result of CD11c expression in three colonic DC subsets is shown (*n* = 4 per each genotype). **d** The number of colonic CD45^+^ CD3^-^B220^-^CD11c^+^ DCs is shown (*n* = 9 for *Ripk1*^kd/kd^ mice and *n* = 13 for CD11c;*Ripk1*^kd/kd^ mice). **e** Representative flow cytometry plots of colonic CD45^+^ cells are shown (left panels, *n* = 4 mice). The number of three colonic DC subsets is shown in the graph on the right (*n* = 2 per each genotype. A representative result from two independent experiments is shown.). **f** Percentages of CD3^−^CD19^−^CD11c^+^ DCs among CD45^+^ cells are shown (*n* = 4 for *Ripk1*^+/+^ mice and *n* = 5 for *Ripk1*^kd/kd^ mice). **g** Representative flow cytometry plots of mLN CD45^+^ cells are shown (*n* = 3 mice). Percentages of DC subsets among CD45^+^ cells are shown in the graph on the right (*n* = 3 per each genotype). Mice from the intercross between *Ripk1*^+/-^ mice were used in (**f**). Mice from the intercross between *Ripk1*^kd/kd^ and CD11c;*Ripk1*^kd/kd^ mice were used in others. Results are mean ± SEM. **p < 0.01 (unpaired *t* test with Welch’s correction).

In the colon, DCs are divided into three groups: CD103^+^ CD11b^−^, CD103^+^ CD11b^+^, and CD103^−^CD11b^+^ DCs. These cells coordinately control colonic immune homeostasis, allowing an optimal response to commensal bacteria and food antigens^32^. RIPK1 was equally expressed in these three subsets (Fig. 2a). While RIPK1 was deleted in CD103^+^ CD11b^−^ and CD103^+^ CD11b^+^ DCs in CD11c;*Ripk1*^kd/kd^ mice, it was still expressed in CD103^−^CD11b^+^ DCs (Fig. 2b). This is most likely due to much lower CD11c expression in CD103^−^CD11b^+^ DCs than in the other two subsets (Fig. 2c). Unlike in the spleen, the number of DCs was significantly reduced in the colon (Fig. 2d). Consistent with the efficiency of RIPK1 deletion (Fig. 2b), the number of CD103^+^ CD11b^−^ and CD103^+^ CD11b^+^ DCs were more strongly reduced than that of CD103^−^CD11b^+^ DCs (Fig. 2e). In contrast, the number of DCs in the colon of *Ripk1*^kd/kd^ mice was normal compared to *Ripk1*^+/+^ mice (Fig. 2f). Because Ki67^high^ population among colonic CD11c^+^ DCs was normal in CD11c;*Ripk1*^kd/kd^ mice (Fig. S3), the decreased number of colonic DCs was not due to impaired proliferation. In addition, CD103^+^ DCs in the mesenteric lymph nodes (mLNs) were decreased in CD11c;*Ripk1*^kd/kd^ mice (Fig. 2g). Because mLN CD103^+^ DCs are migratory DCs from the intestine, this could be partly attributed to the decrease of colonic CD103^+^ DCs in CD11c;*Ripk1*^kd/kd^ mice. In contrast, mLN CD8α^+^ resident DCs were not changed in CD11c;*Ripk1*^kd/kd^ mice, which is similar to the spleen. Furthermore, we found that DCs in the thymus, but not the lung, was decreased in CD11c;*Ripk1*^kd/kd^ mice (Fig. S4). These results indicate that RIPK1 maintains DC number in a tissue-specific manner.

### DC-specific deletion of RIPK1 causes spontaneous colonic inflammation

**Fig. 3:**
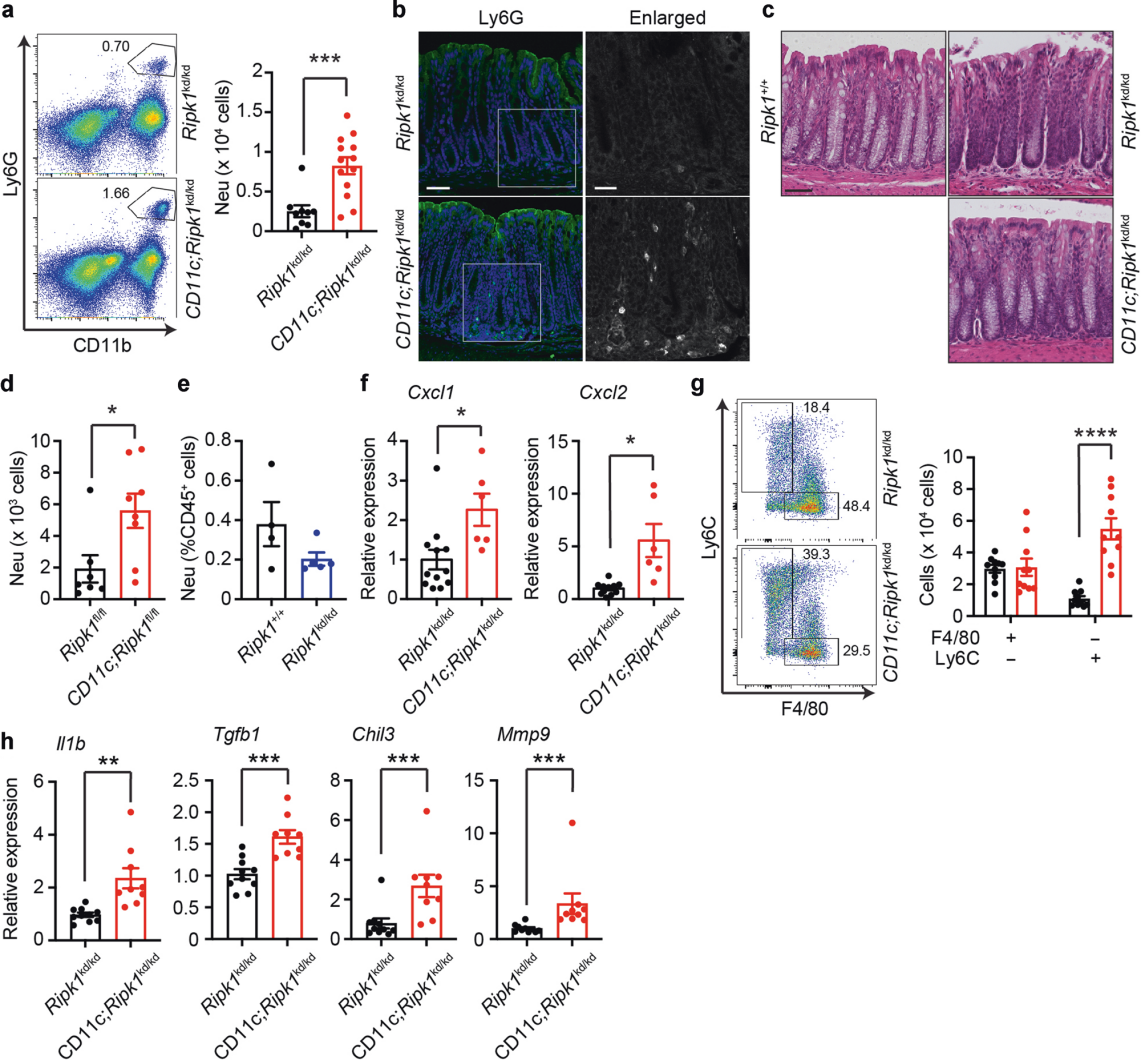
DC-specific RIPK1 deletion causes spontaneous inflammation in the colon. **a** The number of CD45^+^ CD3^−^B220^−^CD11c^-^CD11b^+^ Ly6G^+^ neutrophils (Neu) in the colon is shown (*n* = 9 for *Ripk1*^kd/kd^ mice and *n* = 13 for CD11c;*Ripk1*^kd/kd^ mice). **b** The colon was stained for Ly6G (green) (*n* = 2 per each genotype). The areas indicated by the white squares are enlarged and shown on the right. Nuclei (blue) were stained with DAPI. Scale bars: 50 µm (left panel) and 25 µm (right panel). **c** H&E staining of the colon from mice with the indicated genotypes (*n* = 3 per each genotype). Scale bars: 50 µm. **d** The number of colonic CD45^+^ CD3^−^CD19^−^CD11c^−^CD11b^+^ Ly6G^+^ neutrophils (Neu) is shown. Results are mean ± SEM (*n* = 7 for *Ripk1*^fl/fl^ mice and *n* = 8 for CD11c;*Ripk1*^fl/fl^ mice). **e** Percentages of CD3^−^CD19^−^CD11c^−^CD11b^+^ Ly6G^+^ neutrophils (Neu) among CD45^+^ cells are shown. Results are mean ± SEM (*n* = 4 for *Ripk1*^+/+^ mice and *n* = 5 for *Ripk1*^kd/kd^ mice). **f** The expression of *Cxcl1* and *Cxcl2* in the colon was determined by real-time qPCR. Results are mean ± SEM (*n* = 12 for *Ripk1*^kd/kd^ mice and *n* = 6 for CD11c;*Ripk1*^kd/kd^ mice). **g** The number of F4/80^+^Ly6C^−^ macrophages and F4/80^−^Ly6C^+^ monocytes among CD45^+^ CD3^−^CD19^−^CD11c^−^CD11b^+^ Ly6G^−^ Mono/Mφ in the colon is shown (*n* = 10 per each genotype). **h** Gene expression in sorted colonic lamina propria CD45^+^ CD3^−^CD19^−^CD11c^−^CD11b^+^ Ly6G^−^ Mono/Mφ was determined by real-time PCR. Mice from intercross between either *Ripk1*^fl/fl^ and CD11c;*Ripk1*^fl/fl^ mice (**d**), *Ripk1*^+/−^ mice (**e**), or *Ripk1*^kd/kd^ and CD11c;*Ripk1*^kd/kd^ mice (**a**–**c**, **f**–**h**). *p < 0.05, **p < 0.01, ***p < 0.001, ****p < 0.0001 (unpaired *t* test with Welch’s correction).

As in the spleen, we found increased number of neutrophils in the colon of CD11c;*Ripk1*^kd/kd^ mice, although microscopically visible tissue damage was not observed (Fig. 3a–c). The increased colonic neutrophil was also observed in CD11c;*Ripk1*^fl/fl^ mice (Fig. 3d). In contrast, there was no increase in neutrophils in the colon of *Ripk1*^kd/kd^ mice compared to *Ripk1*^+/+^ mice (Fig. 3e). In addition, neutrophil chemokines *Cxcl1* and *Cxcl2* were highly expressed in the colon of CD11c;*Ripk1*^kd/kd^ mice (Fig. 3f). As in the spleen, we found that CD11b^+^ Ly6G^−^Ly6C^+^ monocytes were significantly increased in the colon of CD11c;*Ripk1*^kd/kd^ mice (Fig. 3g). Unlike in the spleen, Ki67^high^ population in the colonic neutrophils and Mono/Mφ was not changed in CD11c;*Ripk1*^fl/fl^ mice (Fig. S5). Ly6C^+^ monocytes are known to be heterogeneous cell population including pro- and anti-inflammatory cells^33^. For further characterization, we sorted CD11b^+^ Ly6G^−^ Mono/Mφ from *Ripk1*^kd/kd^ and CD11c;*Ripk1*^kd/kd^ mice and compared their gene expression. Importantly, expression of the pro-inflammatory cytokine *Il1b* and the anti-inflammatory cytokine *Tgfb1* was both increased in CD11c;*Ripk1*^kd/kd^ mice (Fig. 3h and S6). In addition, the expression of *Chil3*, which encodes the Ym1 protein, was significantly increased in CD11c;*Ripk1*^kd/kd^ mice. CD11b^+^ Ly6G^−^Ly6C^+^Ym1^+^ monocytes have been reported to have immunoregulatory and tissue-reparative functions^34^. *Mmp9*, which was reported to be highly expressed in the Ym1^+^ monocytes, was also significantly increased in CD11c;*Ripk1*^kd/kd^ mice. In contrast, expression of *Arg1*, *Rentla*, and *Mrc1*, all of which are M2 macrophage markers, was moderately decreased in CD11c;*Ripk1*^kd/kd^ mice (Fig. S6). These results suggest that CD11b^+^ Ly6G^−^ Mono/Mφ in the colon of CD11c;*Ripk1*^kd/kd^ mice contain both pro- and anti-inflammatory monocytes.

### Decreased DCs in the colon of CD11c;Ripk1^kd/kd^ mice is dependent on FADD but not RIPK3

**Fig. 4:**
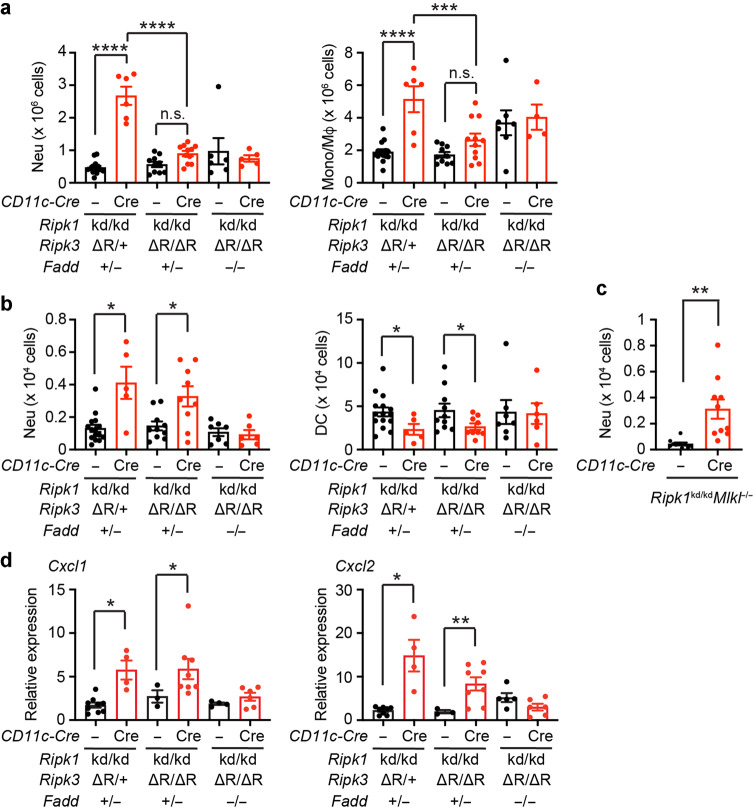
The colonic inflammation in CD11c;*Ripk1*^kd/kd^ mice is restored by deletion of RIPK3 RHIM and FADD. **a**–**c** Splenocytes (**a**) and colonic lamina propria cells (**b**, **c**) isolated from mice with the indicated genotypes were analyzed by flow cytometry. The number of CD45^+^ CD3^−^B220^−^CD11c^−^CD11b^+^ Ly6G^+^ neutrophils (Neu, **a**–**c**), CD45^+^ CD3^−^CD19^−^CD11c^−^CD11b^+^ Ly6G^−^ Mo/Mφ (**a**), CD45^+^ CD3^−^B220^−^CD11c^+^ DCs (**b**) is shown. Results are mean ± SEM (*n* = 5–15 per each genotype). **d** The expression of *Cxcl1* and *Cxcl2* in the colon was determined by real-time qPCR. Results are mean ± SEM (*n* = 3–9 per each genotype). Mice from intercross between *Ripk1*^kd/kd^*Ripk3*^ΔR/ΔR^*Fadd*^−/−^ and CD11c;*Ripk1*^kd/kd^*Ripk3*^ΔR/+^*Fadd*^+/−^ mice were used in (**a**), (**b**), and (**d**). Mice from intercross between *Ripk1*^kd/kd^*Mlkl*^−/−^ and CD11c;*Ripk1*^kd/kd^*Mlkl*^−/−^ mice were used in (**c**). *p < 0.05, **p < 0.01, ***p < 0.001, ****p < 0.0001, n.s.: not significant. (two-way ANOVA in (**a**) and unpaired *t* test with Welch’s correction in others).

Deletion of RIPK1 sensitizes cells to FADD/Caspase 8-dependent apoptosis and RIPK3/MLKL-dependent necroptosis. To examine whether these molecules contribute to the spontaneous inflammation caused by DC-specific RIPK1 deletion, we crossed CD11c;*Ripk1*^kd/kd^ mice with RIPK3 RHIM-deleted mice (*Ripk3*^ΔR/ΔR^ mice)^28^ and/or *Fadd*^−/−^ mice. *Ripk3*^ΔR/ΔR^ mice are resistant to necroptosis because the RHIM is an essential motif for necroptosis induction^28^. Although *Fadd*^−/−^ mice are embryonic lethal due to excessive necroptosis^35^, they are viable in the *Ripk3*^ΔR/ΔR^ genetic background^28^. In the spleen, the increased number of neutrophils and Mono/Mφ was almost completely restored by deletion of RIPK3 RHIM alone (Fig. 4a, 2nd vs 4th bars). In contrast, the number of neutrophils and DCs in the colon was restored by deletion of both FADD and RIPK3 RHIM but not either RIPK3 RHIM or MLKL alone (Fig. 4b, c). Similarly, the increased expression of neutrophil chemokines *Cxcl1* and *Cxcl2* was restored only when FADD and RIPK3 RHIM were both deleted (Fig. 4d).

ZBP1 is a RHIM-containing protein that induces necroptosis through RHIM-dependent interaction with RIPK3 upon recognition of endogenous or viral Z-RNA^36–38^. A recent study revealed that ZBP1 also contributes to RIPK1-dependent caspase 8 activation^39^. RIPK1 deletion triggers ZBP1 and RIPK3-dependent necroptosis. As such, RIPK1 has a critical role in blocking ZBP1-RIPK3 interaction and necroptosis^12,13^. In the absence of ZBP1, the number of splenic neutrophils in CD11c;*Ripk1*^kd/kd^ mice was similar to that in *Ripk1*^kd/kd^ mice (Fig. S7a). In contrast, the increased neutrophil in the colon of CD11c;*Ripk1*^kd/kd^ mice was not reversed by ZBP1 deletion, although the difference in cell number was not statistically different between CD11c;*Ripk1*^kd/kd^;*Zbp1*^−/−^ and *Ripk1*^kd/kd^;*Zbp1*^−/−^ mice (Fig. S7b).

### DC-specific deletion of RIPK1 confers protection against DSS-induced colitis

**Fig. 5:**
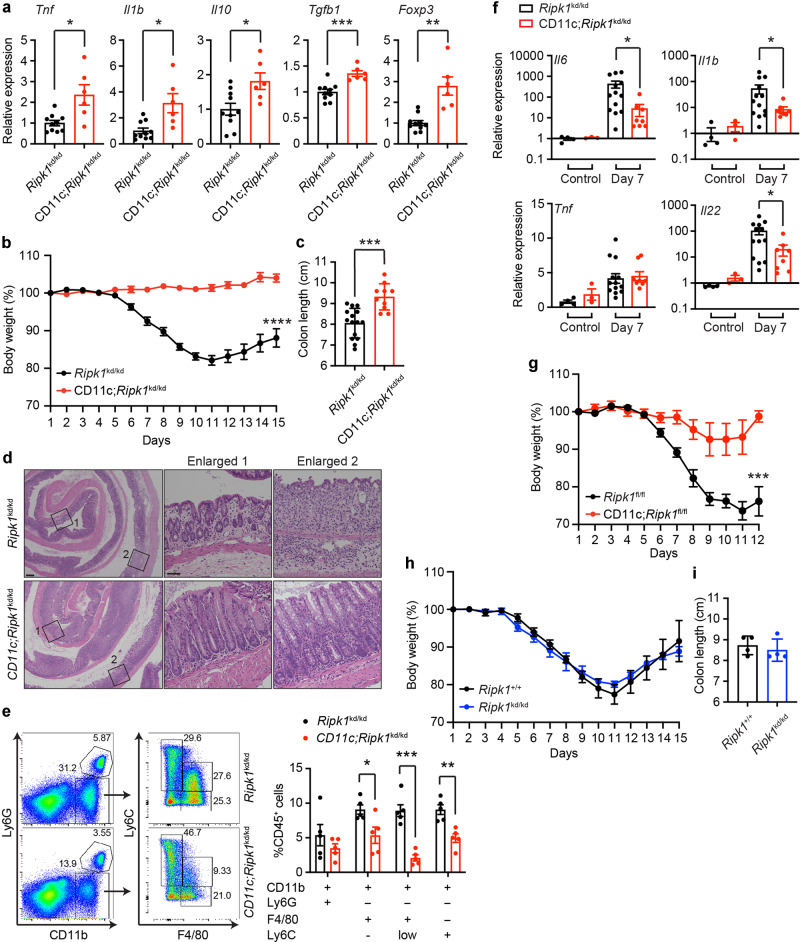
DC-specific deletion of RIPK1 confers protection against DSS-induced colitis. **a** Gene expression in the colon was determined by real-time PCR. Results are mean ± SEM (*n* = 10 for *Ripk1*^kd/kd^ mice and *n* = 6 for CD11c;*Ripk1*^kd/kd^ mice). **b**, **c** Mice with indicated genotypes were treated with DSS for seven days, followed by regular water for an additional seven days. The weight at the beginning of the experiments was normalized as 100%. Daily body weight change (**b**) and colon length on day 15 (**c**) are shown. Results are mean ± SEM (*n* = 15 for *Ripk1*^kd/kd^ mice and *n* = 10 for CD11c;*Ripk1*^kd/kd^ mice). **d** H&E staining of the colon from DSS-treated mice with indicated genotypes (day 7) (*n* = 5 per each genotype). The areas indicated by the black squares on the left (scale bars = 200 µm) were enlarged and shown in the panels on the right (scale bars = 50 µm). **e** Representative flow cytometry plots of CD45^+^ cells in the colon of the mice treated with DSS for seven days are shown. The percentages of each cell subset among CD45^+^ cells are shown in the graph on the right (*n* = 5 per each genotype). **f** The expression of *Il6*, *Il1b*, *Tnf*, and *Il22* in the colon was determined by real-time PCR. Results are mean ± SEM (*n* = 3–13 per each genotype). **g**, **h** Body weight and colon length at day 15 of DSS-treated mice with indicated genotypes are shown. Results are mean ± SEM (*n* = 10 for *Ripk1*^fl/fl^ mice and *n* = 7 for CD11c;*Ripk1*^fl/fl^ mice in (**g**), *n* = 4 per each genotype in (**h**, **i**)). Mice from intercross between either *Ripk1*^fl/fl^ and CD11c;*Ripk1*^fl/fl^ mice (**g**), *Ripk1*^+/−^ mice (**h**, **i**), or *Ripk1*^kd/kd^ and CD11c;*Ripk1*^kd/kd^ mice (**a**–**f**). *p < 0.05, **p < 0.01, ***p < 0.001, ****p < 0.0001 (two-way ANOVA in (**b**, **g**) and unpaired *t* test with Welch’s correction in others).

Consistent with the increase of Mono/Mφ with pro- and anti-inflammatory characteristics, we found that expression of pro-inflammatory cytokines such as *Tnf* and *Il1b* as well as anti-inflammatory cytokines such as *Il10* and *Tgfb1* was significantly increased in the colon of CD11c;*Ripk1*^kd/kd^ mice (Fig. 5a and S8). In addition, expression of the regulatory T cell transcription factor *Foxp3* was also increased in CD11c;*Ripk1*^kd/kd^ mice (Fig. 5a). To examine how this basal colonic immune dysregulation by DC-specific RIPK1 deletion affects development of colitis, we challenged the mice with dextran sulfate sodium (DSS), which induces acute damage of colonic epithelial cells and colitis-like inflammation. The mice were given 1.5% DSS water for seven days followed by regular water for seven days. During the course of experiment, body weight was measured every day to monitor the severity of colitis. *Ripk1*^kd/kd^ mice gradually lost body weight during the first 11 days but started to recover afterwards (Fig. 5b). In contrast, CD11c;*Ripk1*^kd/kd^ mice were highly resistant to DSS-induced colitis and did not exhibit any body weight loss (Fig. 5b). Colon length was significantly longer in CD11c;*Ripk1*^kd/kd^ mice than *Ripk1*^kd/kd^ mice (Fig. 5c). Histological analysis revealed destructed villi and thickened submucosal layer in the colon of *Ripk1*^kd/kd^ mice but not in CD11c;*Ripk1*^kd/kd^ mice (Fig. 5d). Ly6C^+^ monocytes, which were increased in CD11c;*Ripk1*^kd/kd^ mice before DSS treatment (Fig. 3a, g), and also F4/80^+^ macrophages were significantly decreased in CD11c;*Ripk1*^kd/kd^ mice compared to *Ripk1*^kd/kd^ mice after DSS treatment (Fig. 5e). Expression of inflammatory cytokines such as *Il6* and *Il1b*, but not *Tnf*, was significantly lower in CD11c;*Ripk1*^kd/kd^ mice than *Ripk1*^kd/kd^ mice at day 7 (Fig. 5f). Expression of *Il22*, which is a critical cytokine for tissue repair against DSS-induced colitis^40^, was also lower in CD11c;*Ripk1*^kd/kd^ mice. This diminished *Il22* expression is consistent with reduced tissue injury in CD11c;*Ripk1*^kd/kd^ mice. In addition, the protection against DSS-induced colitis was also observed in CD11c;*Ripk1*^fl/fl^ mice (Fig. 5g). Since *Ripk1*^fl/fl^ mice were more sensitive to DSS-induced colitis, several mice had to be euthanized before day 15. Nonetheless, CD11c;*Ripk1*^fl/fl^ littermates were significantly more resistant to DSS-induced colitis than *Ripk1*^fl/fl^ mice, indicating that expression of kinase inactive RIPK1 in non-DC cell types did not contribute to the resistance to DSS. Loss of body weight and colon length at day 15 in *Ripk1*^kd/kd^ mice was similar to that in *Ripk1*^+/+^ mice (Fig. 5h, i), indicating that RIPK1 kinase activity does not affect sensitivity to DSS-induced damage. To examine if the basal increase of neutrophils contributed to the protection against DSS-induced colitis in CD11c;*Ripk1*^kd/kd^ mice, we depleted neutrophils by injecting rat anti-Ly6G antibody in combination with mouse IgG2a anti-rat antibody (Fig. S9a). This double antibody-based depletion strategy was reported to enhance neutrophil depletion^41^. The antibody treatment successfully depleted neutrophils in CD11c;*Ripk1*^kd/kd^ mice (Fig. S9b). However, colon length and pro-inflammatory cytokine expression were not rescued (Fig. S9c, d). Therefore, the increased Ly6G neutrophils does not contribute to the protection in CD11c;*Ripk1*^kd/kd^ mice against DSS-induced colitis. These results indicate that the scaffold-dependent but kinase-independent function of RIPK1 in DCs promotes DSS-induced colitis.

### The protective effect of DC-specific RIPK1 deletion on DSS-induced colitis is mediated by FADD

**Fig. 6:**
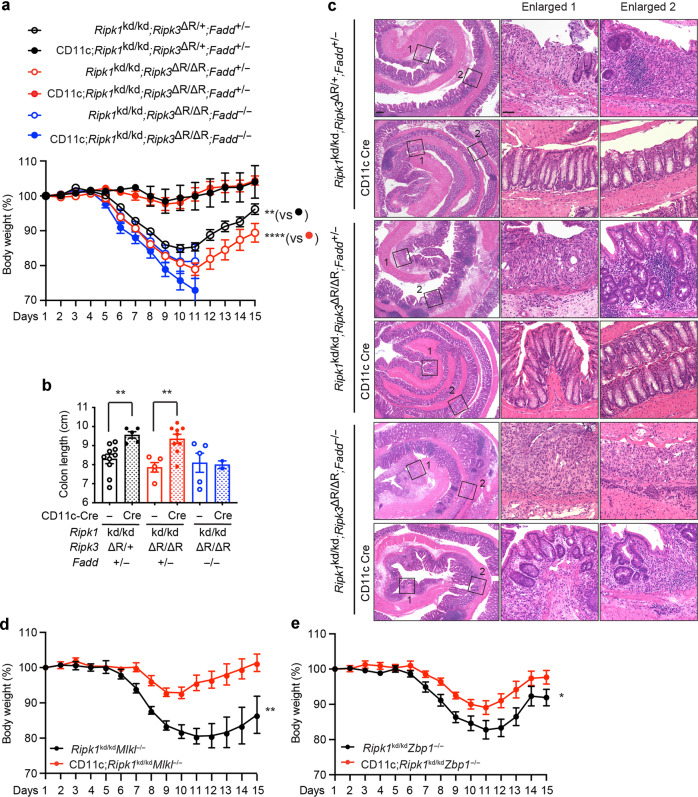
The protective effect of DC-specific RIPK1 deletion in DSS-induced colitis is abrogated by deletion of RIPK3 RHIM and FADD. **a**, **b** Mice with indicated genotypes were treated with DSS for seven days, followed by regular water for an additional seven days. The weight at the beginning of the experiments was normalized as 100%. Daily body weight (**a**) and colon length at day 15 (**b**) are shown. Results are mean ± SEM (*n* = 10 (**a**, **b**) for *Ripk1*^kd/kd^*Ripk3*^ΔR/+^*Fadd*^+/−^ mice, *n* = 5 (**a**, **b**) for CD11c;*Ripk1*^kd/kd^*Ripk3*^ΔR/+^*Fadd*^+/−^ mice, *n* = 5 (**a**, **b**) for *Ripk1*^kd/kd^*Ripk3*^ΔR/ΔR^*Fadd*^+/−^ mice, *n* = 9 (**a**, **b**) for *Ripk1*^kd/kd^*Ripk3*^ΔR/ΔR^*Fadd*^+/−^ mice, *n* = 6 (**a**) and *n* = 5 (**b**) for CD11c;*Ripk1*^kd/kd^*Ripk3*^ΔR/ΔR^*Fadd*^−/−^ mice, *n* = 6 (**a**) and 2 (**b**) for CD11c;*Ripk1*^kd/kd^*Ripk3*^ΔR/ΔR^*Fadd*^−/−^ mice). **c** H&E staining of the colon from DSS-treated mice with indicated genotypes (day 15) (*n* = 2 per each genotype). The areas indicated by the black squares are enlarged and shown in the panels on the right. Scale bars: 200 µm (left panel) and 50 µm (enlarged pictures). **d**, **e** Body weight of DSS-treated mice with indicated genotypes is shown. Results are mean ± SEM (*n* = 7 for *Ripk1*^kd/kd^*Mlkl*^−/−^, *n* = 9 for CD11c;*Ripk1*^kd/kd^*Mlkl*^−/−^, *n* = 11 for *Ripk1*^kd/kd^*Zbp1*^−/−^, and *n* = 13 for CD11c;*Ripk1*^kd/kd^*Zbp1*^−/−^ mice). Mice from intercross between *Ripk1*^kd/kd^*Ripk3*^ΔR/ΔR^*Fadd*^−/−^ and CD11c;*Ripk1*^kd/kd^*Ripk3*^ΔR/+^*Fadd*^+/−^ mice were used in (**a**–**c**). Mice from intercross between *Ripk1*^kd/kd^*Mlkl*^−/−^ and CD11c;*Ripk1*^kd/kd^*Mlkl*^−/−^ mice were used in (**d**). Mice from the intercross between *Ripk1*^kd/kd^*Zbp1*^−/−^ and CD11c;*Ripk1*^kd/kd^*Zbp1*^−/−^ mice were used in (**e**). *p < 0.05, **p < 0.01, ****p < 0.0001 (two-way ANOVA in (**a**, **d**, **e**) and unpaired *t* test with Welch’s correction in (**b**)).

We examined whether apoptosis and/or necroptosis was involved in the protective effect of DC-specific deletion of RIPK1 on DSS-induced colitis. Similar to CD11c;*Ripk1*^kd/kd^ mice (Fig. 5b), CD11c;*Ripk1*^kd/kd^;*Ripk3*^ΔR/+^;*Fadd*^+/-^mice were highly resistant to DSS-induced colitis (Fig. 6a–c). CD11c;*Ripk1*^kd/kd^;*Ripk3*^ΔR/ΔR^;*Fadd*^+/−^ mice were also significantly protected from DSS-induced colitis compared with *Ripk1*^kd/kd^;*Ripk3*^ΔR/ ΔR^;*Fadd*^+/−^mice (Fig. 6a–c). We further found that body weight loss of CD11c;*Ripk1*^kd/kd^;*Mlkl*^−/−^ mice was significantly reduced compared to *Ripk1*^kd/kd^;*Mlkl*^−/−^ mice (Fig. 6d). These results indicate that necroptosis does not contribute to the protective effect of DC-specific RIPK1 deletion on DSS-induced colitis. In contrast, CD11c;*Ripk1*^kd/kd^;*Ripk3*^ΔR/ΔR^;*Fadd*^−/−^ and *Ripk1*^kd/kd^;*Ripk3*^ΔR/ΔR^;*Fadd*^−/−^ mice exhibited similar body weight loss and colonic inflammation in response to DSS (Fig. 6a–c). In fact, the weight loss of both *Ripk1*^kd/kd^;*Ripk3*^ΔR/ΔR^;*Fadd*^−/−^ and CD11c;*Ripk1*^kd/kd^;*Ripk3*^ΔR/ΔR^;*Fadd*^−/−^ mice were severe enough that we needed to euthanize some of the mice before day 15 (Fig. 6a). These results indicate that FADD mediates protection against DSS-induced colitis in CD11c;*Ripk1*^kd/kd^ mice. Deletion of *Zbp1* partially restored sensitivity to DSS in CD11c;*Ripk1*^kd/kd^;*Zbp1*^−/−^ mice, although these mice were still significantly protected from DSS when compared to *Ripk1*^kd/kd^;*Zbp1*^−/−^ mice (Fig. 6e), suggesting that ZBP1 might have a minor contribution to the protective effect of DC-specific RIPK1 deletion on DSS-induced colitis. Collectively, these results indicate that DC-specific RIPK1 deletion confers the protection against DSS-induced colitis in a FADD-dependent but RIPK3-MLKL-independent manner.

## Discussion

In this study, we showed that CD11c;*Ripk1*^kd/kd^ mice, which lack RIPK1 in DCs and RIPK1 kinase activity in other cell types, exhibited spontaneous colonic inflammation characterized by increased neutrophil and Ly6C^+^ monocytes. The fact that *Ripk1*^kd/kd^ mice did not exhibit colonic inflammation indicates that RIPK1 kinase activity was not involved. Despite the spontaneous inflammation, CD11c;*Ripk1*^kd/kd^ mice did not develop gross intestinal tissue destruction and diarrhea, which are characteristics of the patients with *RIPK1* deficiency. Previous studies demonstrated that mice transplanted with *Ripk1*^−/−^ fetal liver cells and hematopoietic cell-specific *Ripk1* knockout mice also did not exhibit intestinal tissue destruction^11,22^. These results suggested that additional factors are required to develop inflammatory bowel disease in mice. However, CD11c;*Ripk1*^kd/kd^ mice were unexpectedly refractory to the colitis when challenged by the colitogenic agent DSS. Previous studies showed that adoptive DC transfer worsened DSS-induced colitis and that diphtheria toxin (DT)-induced deletion of DCs in different types of DT receptor mice ameliorated DSS-induced colitis^42–45^. These studies highlight the role of DCs in promoting colitis. However, it should be noted that there are studies that showed a protective role of DCs in experimental colitis^46,47^. These discrepant results may suggest context-dependent role of DCs in intestinal inflammation. Alternatively, differences in commensal microbiota could contribute to the distinct outcomes. The blunted body weight loss, reduced expression of pro-inflammatory cytokines and the tissue repair cytokine *Il22* in CD11c;*Ripk1*^kd/kd^ mice suggest that DC-specific deletion of RIPK1 suppressed the early inflammatory reaction in response to DSS-induced injury. We found that expression of *Chil3* and *Mmp9* were significantly increased in colonic Mono/Mφ in CD11c;*Ripk1*^kd/kd^ mice, suggesting the presence of Ly6C^+^Ym1^+^ regulatory monocytes. Ly6C^+^Ym1^+^ regulatory monocytes have been reported to be recruited to the colon after tissue injury by DSS and play a role in the resolution of inflammation^34^. It is tempting to speculate that the basal inflammatory state in the colon of CD11c;*Ripk1*^kd/kd^ mice might recruit the Ly6C^+^Ym1^+^ regulatory monocytes to the colon to create an anti-inflammatory tissue milieu that protects against DSS-induced colitis.

Several previous studies have demonstrated that depletion of DCs, impaired DC development, or DC dysfunction could lead to myeloproliferative phenotypes^48–55^, indicating that DCs are a critical cell type that regulates peripheral myeloid cell homeostasis. Although the precise mechanism underlying the myeloproliferation in the absence of intact DCs remains unknown, increased myeloid differentiation has been suggested as one possible mechanism^24,50,53,55^. We found that highly proliferative Ly6G^low^ cells, which could be neutrophil precursors^56,57^, were increased in the spleen and BM of CD11c;*Ripk1*^kd/kd^ mice. In addition, we detected increased proliferation of neutrophils and Mono/Mφ in the spleen and increased neutrophil chemokines in the colon of CD11c;*Ripk1*^kd/kd^ mice. Therefore, the increase of myeloid cells in CD11c;*Ripk1*^kd/kd^ mice could be induced by multiple mechanisms such as increased differentiation, proliferation, and mobilization.

DC-specific-deletion of RIPK1 caused a significant decrease in the number of DCs in the colon, mLN, and thymus. RIPK1 deletion sensitizes cells to cell death in response to cytokines such as TNF and IFNs and pathogen-associated molecular patterns such as LPS^10,11^, all of which are present in abundance in the colon. A very recent study showed that colonic DCs migrate into the thymus and contribute to the development of gut microbiota-specific T cells^58^. Therefore, the reduction of thymic DCs might be attributed to DC deficiency in the gut-draining mLN and colon. Since both resident and migrating thymic DCs are known to be important to maintain self-tolerance^59^, the reduction of thymic DCs might also contribute to the systemic autoimmunity in CD11c;*Ripk1*^fl/fl^ mice^24^.

In contrast to the colon, mLN, and thymus, the number of DCs were not changed in the spleen and BM of CD11c;*Ripk1*^fl/fl^ mice. Since deletion of RIPK3 and/or FADD rescued myeloproliferation and resistance to DSS in CD11c;*Ripk1*^kd/kd^ mice, one can speculate that enhanced RIPK3 and/or FADD-dependent DC cell death might stimulate myelopoiesis to maintain DC number in these tissues. However, we have not been able to detect increased DC apoptosis and necroptosis in vivo. Therefore, we cannot exclude the possibility that DC-specific loss of RIPK1 causes these phenotypes through cell death-independent mechanisms that involves RIPK3 and FADD.

We previously reported that RIPK3 promotes injury-induced cytokine production in DCs in a RHIM-dependent manner. In contrast to *Ripk1* deletion, loss of RIPK3 activity in DCs does not lead to any signs of spontaneous inflammation and that the deletion of RIPK3 RHIM in DCs exacerbated DSS-induced colitis^26–28^. Thus, although RIPK1 and RIPK3 function in synergy to promote necroptosis and detrimental inflammation, they exhibit distinct functions in colonic DCs to regulate intestinal homeostasis. Our study illustrates the multi-functional nature of RIPK1 in controlling life and death and in epithelial tissue homeostasis.

## Methods

### Mice

RIPK1 kinase-dead mutant knock-in mice (*Ripk1*^kd/kd^) in which exon 4 was flanked by loxP sequences were obtained from GlaxoSmithKline^29^. *Ripk3*^ΔR/ΔR^ mice were generated as reported previously^28^. *Fadd*^−/−^ mice were a kind gift from J. Zhang (Thomas Jefferson University). *Mlkl*^−/−^ mice were a kind gift from J. Murphy (Walter Eliza Hall Institute). *Zbp1*^−/−^ mice were obtained from Laboratory of Animal Models for Human Diseases (National Institutes of Biomedical Innovation, Health and Nutrition). CD11c-Cre (*Itgax-cre*) transgenic mice were obtained from Jackson laboratory. *Ripk1*^fl/fl^ mice were a kind gift from M. Pasparakis (University of Cologne). All animals were housed and maintained under specific pathogen-free conditions in the animal facility at Toho University School of Medicine (*Ripk1*^kd/kd^, *Ripk3*^ΔR/ΔR^, *Fadd*^−/−^, and CD11c-Cre mice), Osaka University Graduate School of Medicine (*Ripk1*^kd/kd^, *Ripk3*^ΔR/ΔR^, *Fadd*^−/−^, and CD11c-Cre mice), University of Massachusetts Medical School (*Ripk1*^kd/kd^, *Ripk3*^ΔR/ΔR^, *Fadd*^−/−^, *Mlkl*^−/−^, *Zbp1*^−/−^, *Ripk1*^fl/fl^, and CD11c-Cre mice), and Duke University (*Ripk1*^kd/kd^, *Ripk3*^ΔR/ΔR^, *Fadd*^−/−^, *Mlkl*^−/−^, *Zbp1*^−/−^, *Ripk1*^fl/fl^, and CD11c-Cre mice). All animal experiments were approved by the institutional animal care and use committee.

### Dextran sulfate sodium-induced colitis

DSS (MP Biomedicals, molecular weight 36,000–50,000 Da) was added to sterilized water at a concentration of 1.5% (wt/vol) and administered to age-matched mice (8–12 weeks). After the treatment with DSS for seven days, DSS water was replaced with sterilized water and the mice were sacrificed for analyses at the indicated time point. Body weight was monitored throughout the studies. To deplete neutrophils, InVivoMAb anti-mouse Ly6G antibody (BioXCell, BE0075-1, 50 µg/mouse, once daily) and InVivoMAb anti-rat Kappa immunoglobulin light chain (BioXCell, BE0122, 50 µg/mouse, once every other day) were intraperitoneally injected, starting from three days before DSS treatment. InVivoMAb rat IgG2a isotype control, anti-trinitrophenol (BioXCell, BE0089, 50 µg/mouse, once daily) instead of anti-Ly6G antibody was injected into control mice.

### Histological analysis

The colon was harvested and fixed with 10% formaldehyde, pH 7.0. The tissues were dehydrated and embedded in paraffin. HE staining was performed according to a standard histological procedure. For immunohistochemical staining, fixed tissues were incubated with 20% sucrose in 0.1 M phosphate buffer, pH 7.2 and subsequently frozen in O.C.T. Compound. The frozen tissue sections were treated with blocking solution (5% normal donkey serum in PBS) and then stained with FITC-conjugated anti-Ly6G antibody (BioLegend, 1A8). Nuclei were stained by DAPI. Finally, the tissue slides were mounted with Mowiol mounting medium. Either a BZ-X710 microscope operated by BZ-X Viewer (Keyence) or an FV1000D confocal microscope operated by FluoView software (Olympus) was used to acquire images.

### Flow cytometer

The colon was harvested and incubated in Hanks’ balanced salt solution (HBSS) containing 1 mM DTT for 10 minutes at RT. After incubating in HBSS containing 30 mM EDTA and 10 mM HEPES for 10 minutes at 37 °C twice, the tissues were minced and then incubated in digestion medium (1 mg/ml collagenase IV (Sigma, C5138), 150 µg/ml DNase II (Sigma, DN25), and 0.1 U/ml dispase (STEMCELL Technologies, #07913) in DMEM supplemented with 10% FCS) for 45 minutes at 37 °C. After vigorous shaking followed by filtration, cells were subjected to Percoll gradient centrifugation. The spleen, lung, mLN, and thymus were incubated in collagenase solution (2 mg/ml Collagenase IV, 10 mM HEPES pH 7.4, 150 mM NaCl, 5 mM KCl, 1 mM MgCl_2_, 1.8 mM CaCl_2_) for 30 minutes at 37 °C. BM cells were collected by flushing femurs and tibiae. Splenocytes and BM cells were subjected to erythrocyte lysis (ACK lysis buffer, 150 mM NH_4_Cl, 10 mM KHCO_3_, 100 µM EDTA). Isolated cells were incubated with anti-CD16/32 antibody (2.4G2) and subsequently stained with fluorescent-labeled antibodies shown in Table S1. For intracellular staining of RIPK1, cells stained for surface markers were fixed with 3% paraformaldehyde in PBS for 15 minutes and then permeabilized with 0.05% triton X-100 and 5% normal donkey serum in PBS for 10 minutes. Cells were incubated with anti-RIPK1 antibody (CST, 3493) or control rabbit IgG (Wako, 148-09551) for an hour followed by Alexa Fluor 594-conjugated donkey anti-rabbit IgG (H^+^ L) (Invitrogen) for an hour. For Ki67 staining, cells stained for surface markers were fixed by Foxp3 Fixation & Permeabilization buffer (eBiosciences) for 10 minute and then stained with PE-labeled anti-Ki67 antibody or isotype control IgG for an hour. Cells were analyzed by either FACS CantoII, LSR Fortessa, or FACS Aria III flow cytometers.

### Quantitative PCR

Total RNA was extracted from tissues using NucleoSpin RNA (MACHEREY-NAGEL). cDNA was synthesized using either PrimeScript reverse transcriptase (TAKARA Bio) or ReverTra Ace (TOYOBO). Real-time quantitative PCR was performed with either FastStart Universal Prove Master Mix (Roche Diagnostics) using ViiA 7 Real-Time PCR system (Applied Biosystems), FastStart SYBR Green Master (Roche Diagnostics) using LightCycler 96 (Roche Diagnostics), or Fast SYBR Green Master Mix (Applied Biosystems) using Quant Studio 3 Real-Time PCR system (Applied Biosystems). Glucose-6-phosphate dehydrogenase X-linked (*G6pdx*) and hypoxanthine-guanine phosphoribosyltransferase 1 (*Hprt1*) were used as an internal control. The primers used are shown in Table S2. For *Cxcl1* and *G6pdx*, universal probe 66 and 78 from Roche were used, respectively.

### Generation of BMDCs and ELISA

BMDCs were generated by culturing BM cells from femurs and tibiae in DMEM high glucose medium supplemented with 10% fetal bovine serum, 10 ng/ml GM-CSF (BioLegend, 576306) and 5 ng/ml IL-4 (BioLegend, 574306) for seven days. BMDCs were simulated with ultrapure LPS-EK (Invivogen, tlrl-prklps). Culture medium after stimulation was used for EILSA to detect TNF (BD Biosciences, Opt-EIA, 555268) and IL-6 (BD Biosciences, Opt-EIA, 555240).

### Statistics

Statistical analysis was performed by unpaired *t* test with Welch’s correction, one-way ANOVA, or two-way ANOVA using Prism 8 software. *P* value lower than 0.05 was considered statistically significant.

## Supplementary information

Supplementary Material

## References

[1] Christofferson DE, Li Y, Yuan J (2014). Control of life-or-death decisions by RIP1 kinase. Annu Rev. Physiol..

[2] Liu Z, Chan FK (2021). Regulatory mechanisms of RIPK1 in cell death and inflammation. Semin Cell Dev. Biol..

[3] Delanghe T, Dondelinger Y, Bertrand MJM (2020). RIPK1 kinase-dependent death: a symphony of phosphorylation events. Trends Cell Biol..

[4] Tsuchiya Y, Nakabayashi O, Nakano H (2015). FLIP the switch: regulation of apoptosis and necroptosis by cFLIP. Int J. Mol. Sci..

[5] Chan FK, Luz NF, Moriwaki K (2015). Programmed necrosis in the cross talk of cell death and inflammation. Annu. Rev. Immunol..

[6] Li J (2012). The RIP1/RIP3 necrosome forms a functional amyloid signaling complex required for programmed necrosis. Cell.

[7] Mompean M (2018). The structure of the necrosome RIPK1-RIPK3 core, a human hetero-amyloid signaling complex. Cell.

[8] Upton JW, Kaiser WJ, Mocarski ES (2012). DAI/ZBP1/DLM-1 complexes with RIP3 to mediate virus-induced programmed necrosis that is targeted by murine cytomegalovirus vIRA. Cell Host Microbe.

[9] He S, Liang Y, Shao F, Wang X (2011). Toll-like receptors activate programmed necrosis in macrophages through a receptor-interacting kinase-3-mediated pathway. Proc. Natl Acad. Sci. USA.

[10] Dillon CP (2014). RIPK1 blocks early postnatal lethality mediated by caspase-8 and RIPK3. Cell.

[11] Rickard JA (2014). RIPK1 regulates RIPK3-MLKL-driven systemic inflammation and emergency hematopoiesis. Cell.

[12] Newton K (2016). RIPK1 inhibits ZBP1-driven necroptosis during development. Nature.

[13] Lin J (2016). RIPK1 counteracts ZBP1-mediated necroptosis to inhibit inflammation. Nature.

[14] Degterev A, Ofengeim D, Yuan J (2019). Targeting RIPK1 for the treatment of human diseases. Proc. Natl Acad. Sci. USA.

[15] Mifflin L, Ofengeim D, Yuan J (2020). Receptor-interacting protein kinase 1 (RIPK1) as a therapeutic target. Nat. Rev. Drug Disco..

[16] Kelliher MA (1998). The death domain kinase RIP mediates the TNF-induced NF-kappaB signal. Immunity.

[17] Cuchet-Lourenco D (2018). Biallelic RIPK1 mutations in humans cause severe immunodeficiency, arthritis, and intestinal inflammation. Science.

[18] Li Y (2019). Human RIPK1 deficiency causes combined immunodeficiency and inflammatory bowel diseases. Proc. Natl Acad. Sci. USA.

[19] Uchiyama Y (2019). Primary immunodeficiency with chronic enteropathy and developmental delay in a boy arising from a novel homozygous RIPK1 variant. J. Hum. Genet..

[20] Dannappel M (2014). RIPK1 maintains epithelial homeostasis by inhibiting apoptosis and necroptosis. Nature.

[21] Takahashi N (2014). RIPK1 ensures intestinal homeostasis by protecting the epithelium against apoptosis. Nature.

[22] Roderick JE (2014). Hematopoietic RIPK1 deficiency results in bone marrow failure caused by apoptosis and RIPK3-mediated necroptosis. Proc. Natl Acad. Sci. USA.

[23] Dowling JP, Cai Y, Bertin J, Gough PJ, Zhang J (2016). Kinase-independent function of RIP1, critical for mature T-cell survival and proliferation. Cell Death Dis..

[24] O’Donnell JA (2018). Dendritic cell RIPK1 maintains immune homeostasis by preventing inflammation and autoimmunity. J. Immunol..

[25] Mildner A, Jung S (2014). Development and function of dendritic cell subsets. Immunity.

[26] Moriwaki K (2014). The necroptosis adaptor RIPK3 promotes injury-induced cytokine expression and tissue repair. Immunity.

[27] Moriwaki K, Bertin J, Gough PJ, Chan FKM (2015). A RIPK3-Caspase 8 complex mediates atypical pro-IL-1b processing. J. Immunol..

[28] Moriwaki K, Balaji S, Bertin J, Gough PJ, Chan FK (2017). Distinct kinase-independent role of RIPK3 in CD11c(+) mononuclear phagocytes in cytokine-induced tissue repair. Cell Rep..

[29] Berger SB (2014). Cutting Edge: RIP1 kinase activity is dispensable for normal development but is a key regulator of inflammation in SHARPIN-deficient mice. J. Immunol..

[30] Polykratis A (2014). Cutting edge: RIPK1 Kinase inactive mice are viable and protected from TNF-induced necroptosis in vivo. J. Immunol..

[31] Cusson-Hermance N, Khurana S, Lee TH, Fitzgerald KA, Kelliher MA (2005). Rip1 mediates the Trif-dependent toll-like receptor 3- and 4-induced NF-{kappa}B activation but does not contribute to interferon regulatory factor 3 activation. J. Biol. Chem..

[32] Sun T, Nguyen A, Gommerman JL (2020). Dendritic cell subsets in intestinal immunity and inflammation. J. Immunol..

[33] Guilliams M, Mildner A, Yona S (2018). Developmental and functional heterogeneity of monocytes. Immunity.

[34] Ikeda N (2018). Emergence of immunoregulatory Ym1(+)Ly6C(hi) monocytes during recovery phase of tissue injury. Sci. Immunol..

[35] Zhang H (2011). Functional complementation between FADD and RIP1 in embryos and lymphocytes. Nature.

[36] Maelfait J (2017). Sensing of viral and endogenous RNA by ZBP1/DAI induces necroptosis. Embo J..

[37] Jiao H (2020). Z-nucleic-acid sensing triggers ZBP1-dependent necroptosis and inflammation. Nature.

[38] Zhang T (2020). Influenza virus Z-RNAs induce ZBP1-mediated necroptosis. Cell.

[39] Muendlein HI (2021). ZBP1 promotes LPS-induced cell death and IL-1beta release via RHIM-mediated interactions with RIPK1. Nat. Commun..

[40] Zenewicz LA (2008). Innate and adaptive interleukin-22 protects mice from inflammatory bowel disease. Immunity.

[41] Boivin G (2020). Durable and controlled depletion of neutrophils in mice. Nat. Commun..

[42] Berndt BE, Zhang M, Chen GH, Huffnagle GB, Kao JY (2007). The role of dendritic cells in the development of acute dextran sulfate sodium colitis. J. Immunol..

[43] Arimura K (2017). Crucial role of plasmacytoid dendritic cells in the development of acute colitis through the regulation of intestinal inflammation. Mucosal Immunol..

[44] Abe K (2007). Conventional dendritic cells regulate the outcome of colonic inflammation independently of T cells. Proc. Natl Acad. Sci. USA.

[45] Kourepini E (2014). Osteopontin expression by CD103- dendritic cells drives intestinal inflammation. Proc. Natl Acad. Sci. USA.

[46] Muzaki AR (2016). Intestinal CD103(+)CD11b(-) dendritic cells restrain colitis via IFN-gamma-induced anti-inflammatory response in epithelial cells. Mucosal Immunol..

[47] Qualls JE, Tuna H, Kaplan AM, Cohen DA (2009). Suppression of experimental colitis in mice by CD11c+ dendritic cells. Inflamm. Bowel Dis..

[48] Jiao J (2014). Central role of conventional dendritic cells in regulation of bone marrow release and survival of neutrophils. J. Immunol..

[49] Tittel AP (2012). Functionally relevant neutrophilia in CD11c diphtheria toxin receptor transgenic mice. Nat. Methods.

[50] Birnberg T (2008). Lack of conventional dendritic cells is compatible with normal development and T cell homeostasis, but causes myeloid proliferative syndrome. Immunity.

[51] Holtschke T (1996). Immunodeficiency and chronic myelogenous leukemia-like syndrome in mice with a targeted mutation of the ICSBP gene. Cell.

[52] Turcotte K (2005). A mutation in the Icsbp1 gene causes susceptibility to infection and a chronic myeloid leukemia-like syndrome in BXH-2 mice. J. Exp. Med..

[53] Wang Y (2012). Transforming growth factor beta-activated kinase 1 (TAK1)-dependent checkpoint in the survival of dendritic cells promotes immune homeostasis and function. Proc. Natl Acad. Sci. USA.

[54] Satpathy AT (2014). Runx1 and Cbfbeta regulate the development of Flt3+ dendritic cell progenitors and restrict myeloproliferative disorder. Blood.

[55] Humblet-Baron S (2019). Murine myeloproliferative disorder as a consequence of impaired collaboration between dendritic cells and CD4 T cells. Blood.

[56] Kim MH (2017). A late-lineage murine neutrophil precursor population exhibits dynamic changes during demand-adapted granulopoiesis. Sci. Rep..

[57] Zhu YP (2018). Identification of an early unipotent neutrophil progenitor with pro-tumoral activity in mouse and human bone marrow. Cell Rep..

[58] Zegarra-Ruiz DF (2021). Thymic development of gut-microbiota-specific T cells. Nature.

[59] Audiger C, Rahman MJ, Yun TJ, Tarbell KV, Lesage S (2017). The importance of dendritic cells in maintaining immune tolerance. J. Immunol..

