# Supplementary material for: The scaffold-dependent function of RIPK1 in dendritic cells promotes injury-induced colitis
Source: Mucosal Immunol. 2021 Aug 30;15(1):84–95. doi: 10.1038/s41385-021-00446-y (PMC8732271; doi:10.1038/s41385-021-00446-y)
Supplement: Supplementary file 1 — Supplementary Material [file 41385_2021_446_MOESM1_ESM.pdf]

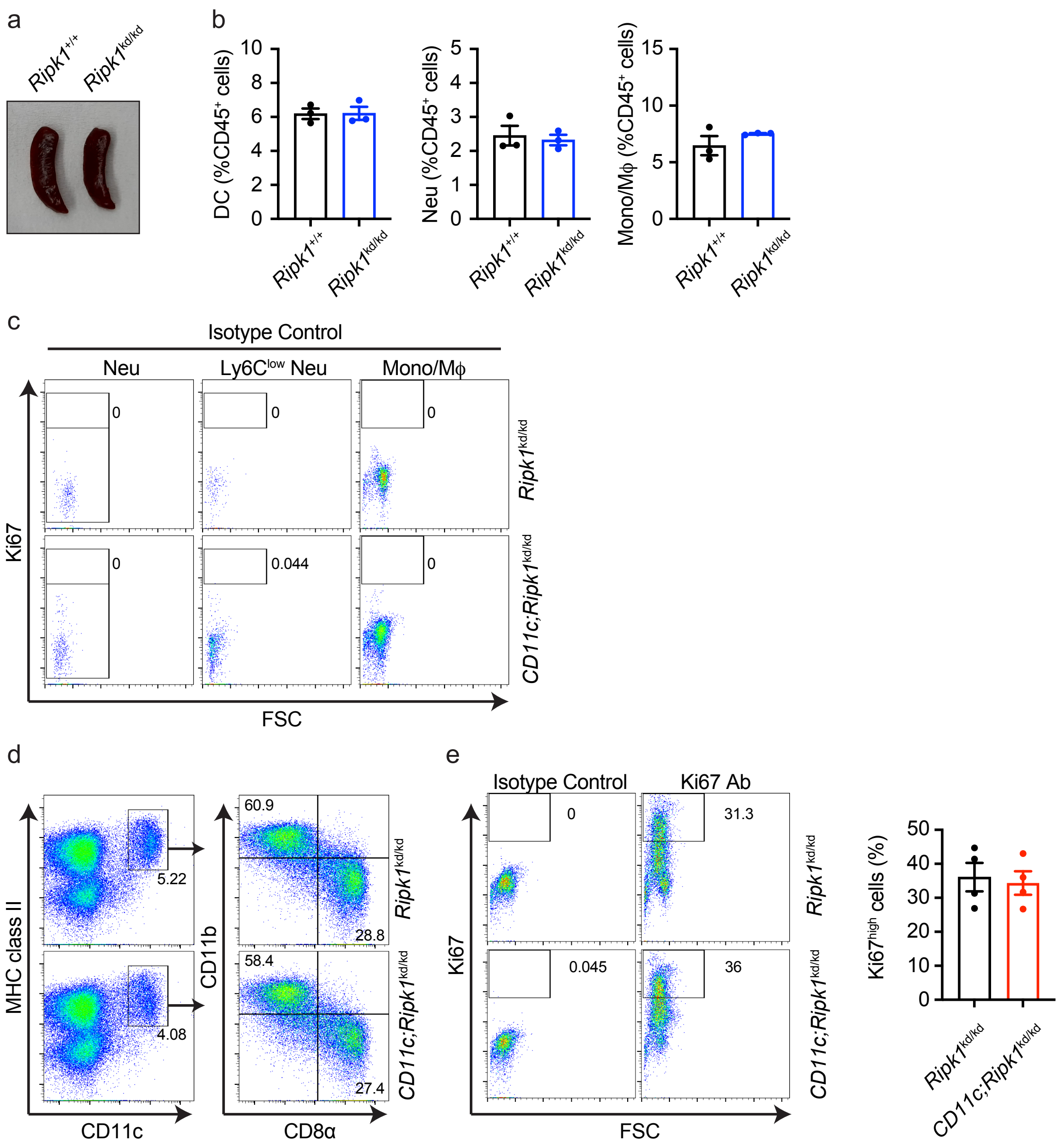

Supplementary Figure 1: Normal immune cell population in *Ripk1*$^{kd/kd}$ mice.

(a) A representative picture of the spleen from *Ripk1*$^{+/+}$ and *Ripk1*$^{kd/kd}$ mice is shown. **(b)** Percentages of CD3^{-}CD19^{-}CD11c^{+} DCs, CD3^{-}CD19^{-}CD11c^{-}CD11b^{+}Ly6G^{+} neutrophils (Neu), and CD3^{-}CD19^{-}CD11c^{-}CD11b^{+}Ly6G^{-} monocytes/macrophages (Mono/Mφ) among CD45^{+} cells are shown (n = 3 per each genotype). **(c)** Representative flow cytometry plots of CD45^{+}TCRβ^{-}CD19^{-}CD11c^{-}CD11b^{+}Ly6G^{+} Neu, CD45^{+}TCRβ^{-}CD19^{-}CD11c^{-}CD11b^{+}Ly6G^{-} Mo/Mφ, and CD45^{+}TCRβ^{-}CD19^{-}CD11c^{-}CD11b^{+}Ly6G^{low} cells in the spleen are shown. The staining was performed using PE-labled isotype control instead of PE-labeld anti-Ki67 antibody. The representative FACS plots of the cells stained by PE-labeled anti-Ki67 antibody are shown in Fig. 1f. **(d)** Representative flow cytometry plots of CD45^{+} cells in the spleen are shown. **(e)** Representative flow cytometry plots of CD45^{+}TCRβ^{-}CD19^{-}CD11c^{+} DCs in the spleen are shown. Percentages of Ki67^{high} population among DCs are shown in the graph on the right (n = 4 per each genotype). Results are mean ± SEM. Mice from the intercross between either *Ripk1*$^{+/-}$ mice **(a, b)** or *Ripk1*$^{kd/kd}$ and CD11c;*Ripk1*$^{kd/kd}$ mice **(c-e)** were used.

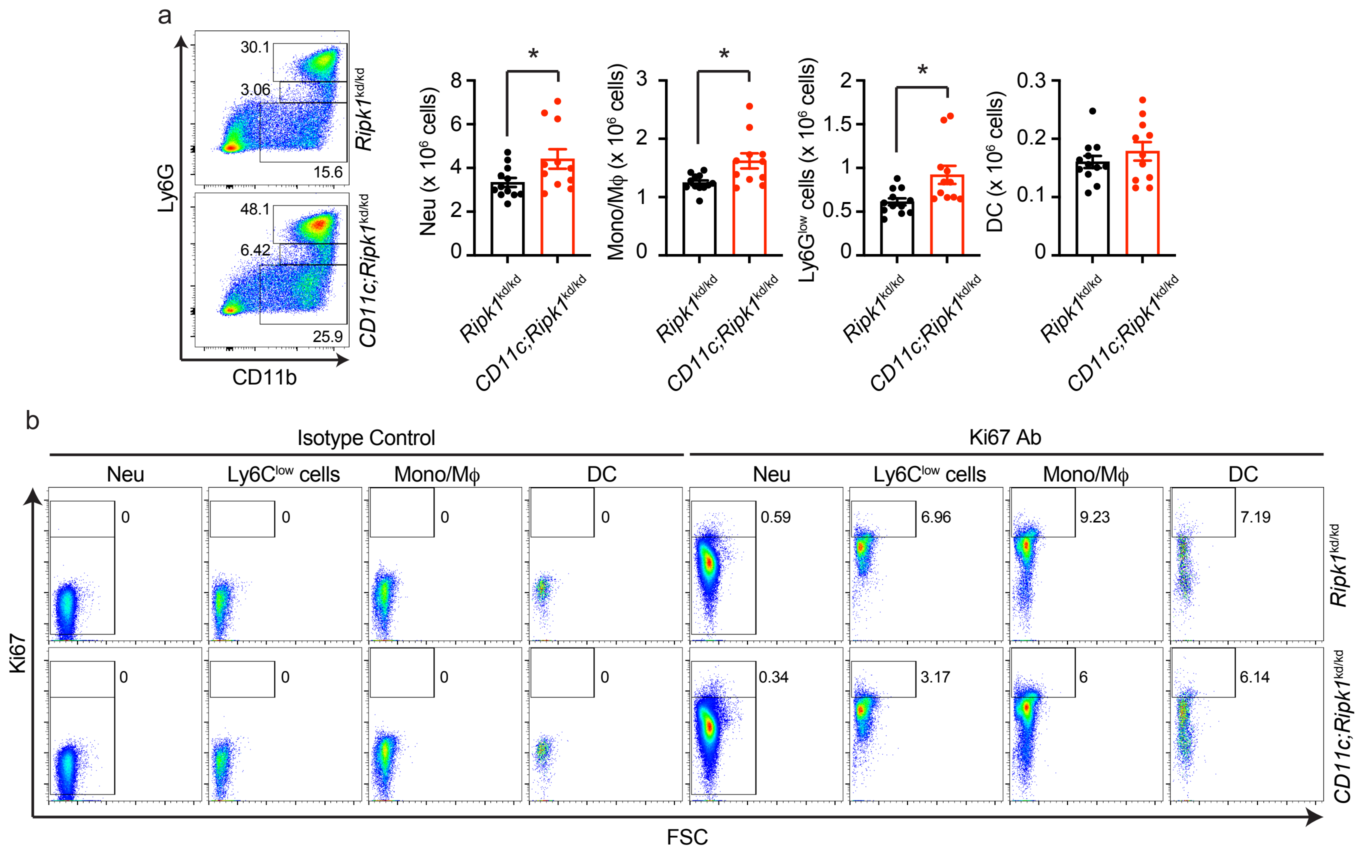

Supplementary Figure 2: Increased neutrophils in the BM of CD11c;*Ripk1*$^{kd/kd}$ mice.
(a) Representative flow cytometry plots of CD45^{+}CD3^{-}B220^{-}CD11c^{-} cells in the BM are shown. The number of CD45^{+}CD3^{-}B220^{-}CD11c^{-}CD11b^{+}Ly6G^{+} neutrophils (Neu), CD45^{+}CD3^{-}B220^{-}CD11c^{-}CD11b^{+}Ly6G^{-} monocytes/macrophages (Mono/Mφ), CD45^{+}CD3^{-}B220^{-}CD11c^{-}CD11b^{+}Ly6G^{low} cells, and CD45^{+}CD3^{-}B220^{-}CD11c^{+} DCs in the BM is shown. Results are mean ± SEM (n = 12 for *Ripk1*$^{kd/kd}$ mice and n = 11 for CD11c;*Ripk1*$^{kd/kd}$ mice). **(b)** BM cells were subjected to Ki67 staining. Representative flow cytometry plots of Neu, Mo/Mφ, Ly6G^{low} cells, and DCs in the BM are shown (n = 4 per each genotype). Mice from the intercross between *Ripk1*$^{kd/kd}$ and CD11c;*Ripk1*$^{kd/kd}$ mice were used. *$p < 0.05$ (unpaired t test with Welch's correction).

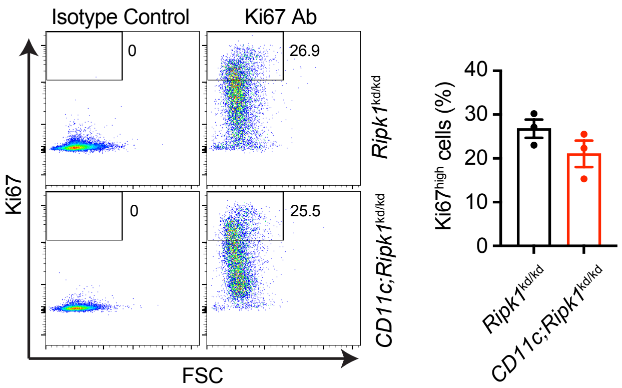

Supplementary Figure 3: Proliferation of colonic DCs in CD11c;*Ripk1*$^{kd/kd}$ mice. Representative flow cytometry plots of CD45^{+}TCRβ^{-}CD19^{-}CD11c^{+} DCs in the colon are shown. Percentages of Ki67^{high} population among DCs are shown in the graph on the right (n = 3 per each genotype). Results are mean ± SEM. Mice from the intercross between *Ripk1*$^{kd/kd}$ and CD11c;*Ripk1*$^{kd/kd}$ mice were used.

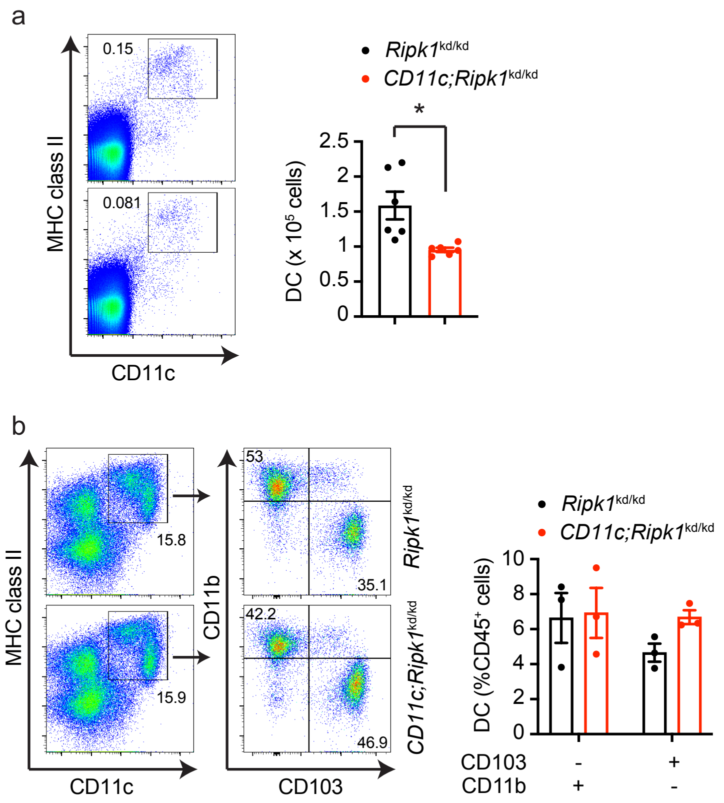

Supplementary Figure 4: DCs in the thymus and lung of CD11c;*Ripk1*$^{kd/kd}$ mice.
Representative flow cytometry plots of CD3$^-$CD19$^-$B220$^-$ cells in the thymus **(a)** and CD45$^+$ cells in the lung **(b)** are shown. The number of CD11c$^+$MHC class II$^+$ DCs in the thymus is shown on the right in **a** (n = 6 per each genotype). Percentages of CD11c$^+$MHC class II$^+$CD103$^+$CD11b$^-$ and CD11c$^+$MHC class II$^+$CD103$^-$CD11b$^+$ DCs among CD45$^+$ cells are shown on the right in **b** (n = 3 per each genotype). Results are mean ± SEM. Mice from the intercross between *Ripk1*$^{kd/kd}$ and CD11c;*Ripk1*$^{kd/kd}$ mice were used. *$p < 0.05$ (unpaired t test with Welch's correction).

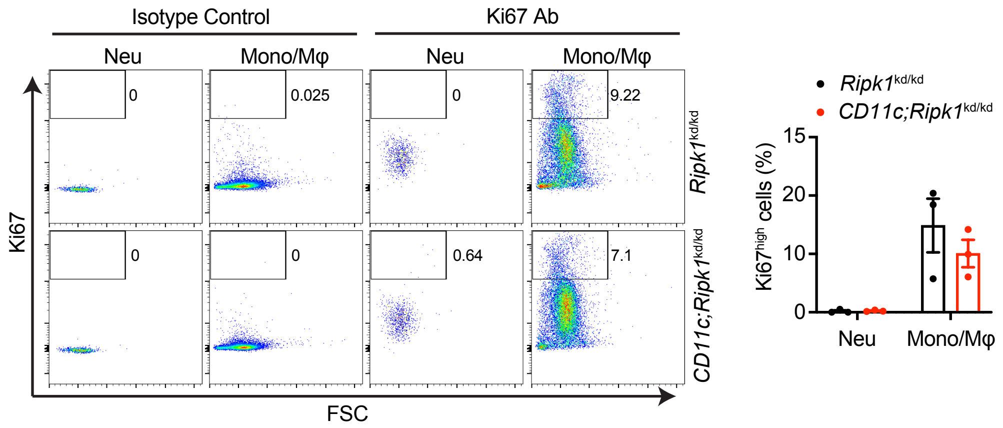

Supplementary Figure 5: Proliferation of colonic neutrophils and monocytes/macrophages in CD11c;*Ripk1*$^{kd/kd}$ mice. Representative flow cytometry plots of CD45^{+}TCRβ^{-}CD19^{-}CD11c^{-}CD11b^{+}Ly6G^{+} neutrophils (Neu) and CD45^{+}TCRβ^{-}CD19^{-}CD11c^{-}CD11b^{+}Ly6G^{-} monocytes/macrophages (Mo/Mφ) in the colon are shown. Percentages of Ki67^{high} population among the indicated cell subsets are shown in the graph on the right (n = 3 per each genotype). Results are mean ± SEM. Mice from the intercross between *Ripk1*$^{kd/kd}$ and CD11c;*Ripk1*$^{kd/kd}$ mice were used.

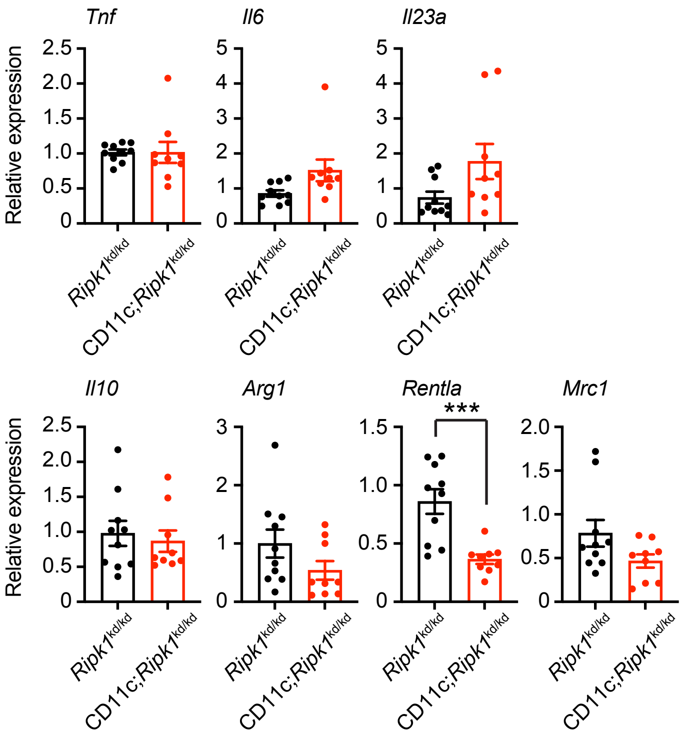

Supplementary Figure 6: Gene expression in the colonic monocytes/macrophages in CD11c;*Ripk1*$^{kd/kd}$ mice.
Gene expression in sorted colonic lamina propria CD45^{+}CD3^{-}CD19^{-}CD11c^{-}CD11b^{+}Ly6G^{-} Mono/Mφ was determined by real-time PCR. Mice from intercross between *Ripk1*$^{kd/kd}$ and CD11c;*Ripk1*$^{kd/kd}$ mice were used. ***$p < 0.001$ (unpaired t test with Welch's correction).

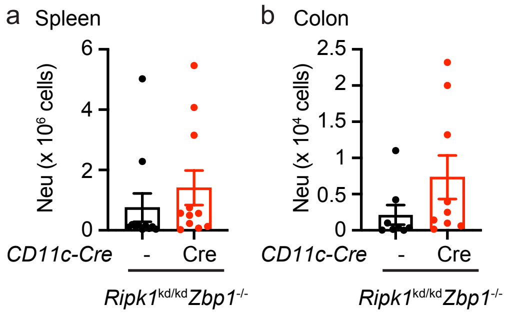

Supplementary Figure 7: The increased colonic neutrophil in CD11c;*Ripk1*$^{kd/kd}$ mice is not restored by deletion of ZBP1. Splenocytes **(a)** and colonic lamina propria cells **(b)** isolated from mice with indicated genotypes were analyzed by flow cytometry. The number of $CD3^{-}CD19^{-}CD11c^{-}CD11b^{+}Ly6G^{+}$ neutrophils (Neu) are shown. Results are mean ± SEM (n = 8 **(a)** and 11 **(b)** for *Ripk1*$^{kd/kd}$*Zbp1*$^{-/-}$ mice and n = 9 **(a)** and 11 **(b)** for CD11c;*Ripk1*$^{kd/kd}$*Zbp1*$^{-/-}$ mice). Mice from intercross between *Ripk1*$^{kd/kd}$*Zbp1*$^{-/-}$ and CD11c;*Ripk1*$^{kd/kd}$*Zbp1*$^{-/-}$ mice were used.

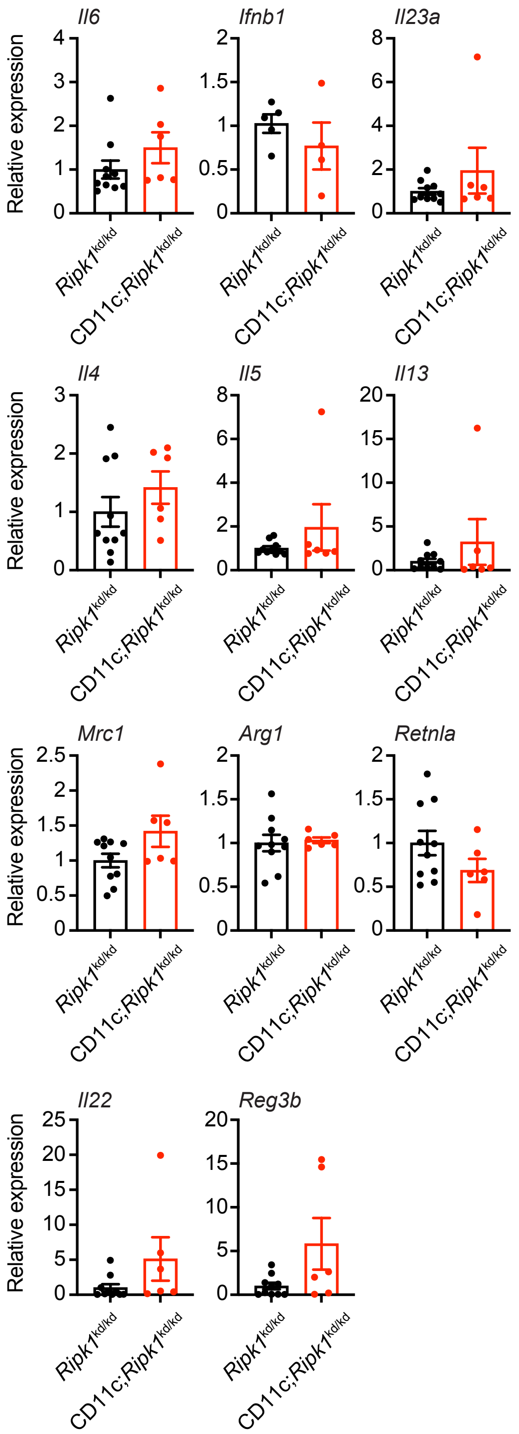

Supplementary Figure 8: Gene expression in the colon of CD11c;*Ripk1*$^{kd/kd}$ mice.

Gene expression in the colon was determined by real-time PCR. Results are mean ± SEM (n = 10 for *Ripk1*$^{kd/kd}$ mice and n = 6 for CD11c;*Ripk1*$^{kd/kd}$ mice). Mice from intercross between *Ripk1*$^{kd/kd}$ and CD11c;*Ripk1*$^{kd/kd}$ mice were used.

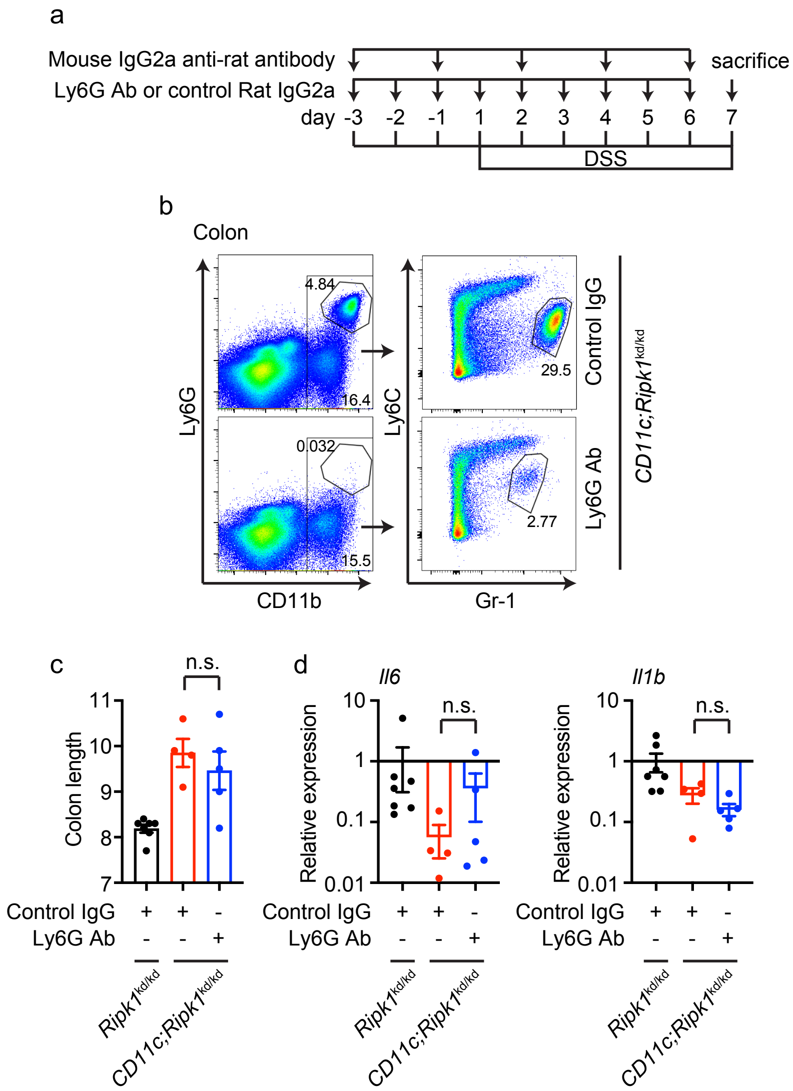

Supplementary Figure 9: Depletion of neutrophils does not affect the resistance of CD11c;*Ripk1*$^{kd/kd}$ mice against DSS-induced colitis. **(a)** Either rat anti-Ly6G antibody or control rat IgG2a was injected every day from three days before DSS administration. Mouse IgG2a anti-rat antibody was injected every other day from three days before DSS administration. **(b)** Representative flow cytometry plots of CD45^{+}cells in the colon of CD11c;*Ripk1*$^{kd/kd}$ mice are shown. Because injected Ly6G antibody masks Ly6G antigen on the cell surface (left panels), neutrophils were detected as CD11b^{+}Gr-1^{+}Ly6C^{int} cells without using Ly6G antibody (right panels). **(c,d)** Colon length **(c)** and inflammatory cytokine gene expression in the colon **(d)** were examined at day 7. n.s.: not significant. Results are mean ± SEM (n = 4-7). Mice from intercross between *Ripk1*$^{kd/kd}$ and CD11c;*Ripk1*$^{kd/kd}$ mice were used.

Supplementary Table 1. A list of antibodies used in this study.

Molecules	Fluorophores	Vendors	Clone
B220	APC Cy7	BioLegend	RA3-6B2
B220	Biotin	BD Biosciences	RA3-6B2
CD103	Biotin	BioLegend	2E7
CD11b	BV421	BD Biosciences	M1/70
CD11b	PerCP Cy5.5	BioLegend	M1/70
CD11c	APC	BD Biosciences	HL3
CD11c	PE	TONBO Biosciences	N418
CD19	PE	TONBO Biosciences	1D3
CD19	PE Cy7	eBiosciences	eBio1D3
CD3e	APC Cy7	BioLegend	145-2C11
CD3e	PE	BioLegend	145-2C11
CD45.2	APC Cy7	BD Biosciences	104
CD45.2	Pacific Blue	BioLegend	104
CD45.2	PE Cy7	BioLegend	104
CD80	PE	eBiosciences	16-10A1
CD86	PE Cy7	eBiosciences	GL1
CD8α	APC Cy7	TONBO Biosciences	53-6.7
CD8α	PE	TONBO Biosciences	53-6.7
F4/80	APC	TONBO Biosciences	BM8.1
F4/80	PE	TONBO Biosciences	BM8.1
Gr-1	APC	TONBO Biosciences	RB6-8C5
Ly6C	APC Cy7	BioLegend	HK1.4
Ly6C	FITC	BioLegend	HK1.4
Ly6G	APC	BioLegend	1A8
Ly6G	FITC	BioLegend	1A8
MHC Class II I-Ab	FITC	eBiosciences	AF-6-120.1
Streptavidin	APC eFluor780	BioLegend	47-4317-82
TCRβ	PE Cy7	TONBO Biosciences	H57-597

Supplementary Table 2. A list of primers used in this study.

**Genes**	**Forward**	**Reverse**
Arg1	CCACACACCTGTAAGCCAGG	CAGTACTTGATGGTCCTTCCG
Chil3	AAAGACAAGAACACTGAGCTAAAAACTC	GAATCTGATAACTGACTGAATGAATATC
Cxcl1	CCACACTCAAGAATGGTCGC	TCTCCGTTACTTGGGGACAC
Cxcl2	AAAATCATCCAAAAGATACTGAACAA	CTTTGGTTCTTCCGTTGAGG
Foxp3	GGCGAAAGTGGCAGAGAGG	AAGGCAGAGTCAGGAGAAGTTG
G6pdx	GAAAGCAGAGTGAGCCCTTC	CATAGGAATTACGGGCAAGA
Hprt1	AACAAAGTCTGGCCTGTATCCAA	GCAGTACAGCCCCAAAATGG
Ifnb1	CAGCTCCAAGAAAGGACGAAC	GGCAGTGTAACTCTTCTGCAT
Il10	TGAAGACCCTCAGGATGCGG	AGACACCTTGGTCTTGGAGCTTA
Il13	GCAGCAAGACCGTGAGTCC	GACTCATTTAAATAACACACAACC
Il1b	GAGCTGAAAGCTCTCCACCTCA	TCGTTGCTTGGTTCTCCTTGTAC
Il22	GCTCCTGTCACATCAGCGGTGAC	GCAGGTCCAGTTCCCCAATCGCC
Il23a	GGGGAACATTATACTTTCCTGG	CTAGATTCTGTTAGAACTGAGG
Il4	AGATGGATGTGCCAAACGTCCTCA	AATATGCGAAGCACCTTGGAAGCC
Il5	ACGCAGGAGGATCACATACC	AATTGAAGTTAGATAGGAGCAGG
Il6	CACAGAGGATACCACTCCCAA	TCCACGATTTCCCAGAGAACA
Mmp9	CTTCCCCAAAGACCTGAAAAC	CTGCTTCTCTCCCATCATCTG
Mrc1	TCTTGTTTGTCCCAGGCAAGG	ACCCAGTTATGCAAATTTACAGG
Reg3b	TGACATGTGAGGTGAAGTTGC	CTTCACATTTTGTCCCTTGTCC
Rentla	ATCTTGGGAGATCCAGAGTGG	TCAAAGCTGGGTTCTCCACC
Tgfb1	GACGGAATACAGGGCTTTCG	ACGTTTGGGGCTGATCCCG
Tnf	GGTGCCTATGTCTCAGCCTCTT	CGATCACCCCGAAGTTCAGTA
